# Human Gb3/CD77 synthase produces P1 glycotope-capped N-glycans, which mediate Shiga toxin 1 but not Shiga toxin 2 cell entry

**DOI:** 10.1016/j.jbc.2021.100299

**Published:** 2021-01-15

**Authors:** Katarzyna Szymczak-Kulus, Sascha Weidler, Anna Bereznicka, Krzysztof Mikolajczyk, Radoslaw Kaczmarek, Bartosz Bednarz, Tao Zhang, Anna Urbaniak, Mariusz Olczak, Enoch Y. Park, Edyta Majorczyk, Katarzyna Kapczynska, Jolanta Lukasiewicz, Manfred Wuhrer, Carlo Unverzagt, Marcin Czerwinski

**Affiliations:** 1 Laboratory of Glycobiology, Hirszfeld Institute of Immunology and Experimental Therapy, Wroclaw, Poland; 2 Department of Bioorganic Chemistry, University of Bayreuth, Bayreuth, Germany; 3 Laboratory of Molecular Biology of Microorganisms, Hirszfeld Institute of Immunology and Experimental Therapy, Wroclaw, Poland; 4 Center for Proteomics and Metabolomics, Leiden University Medical Center, Leiden, The Netherlands; 5 Department of Biochemistry and Molecular Biology, Faculty of Veterinary Medicine, Wroclaw University of Environmental and Life Sciences, Wroclaw, Poland; 6 Department of Biochemistry, Faculty of Biotechnology, University of Wroclaw, Wroclaw, Poland; 7 Laboratory of Biotechnology, Shizuoka University, Shizuoka, Japan; 8 Faculty of Physical Education and Physiotherapy, Opole University of Technology, Opole, Poland; 9 Laboratory of Medical Microbiology, Hirszfeld Institute of Immunology and Experimental Therapy, Wroclaw, Poland; 10 Laboratory of Microbial Immunochemistry and Vaccines, Hirszfeld Institute of Immunology and Experimental Therapy, Wroclaw, Poland

**Keywords:** N-glycan, glycoprotein, glycolipid, Shiga toxin, receptor, glycosyltransferase, A4GALT, glycosylation inhibitor, globotriaosylceramide (Gb3), P1 antigen, Band 3, Band 3 anion transport protein, ESI-MS, electrospray ionisation–mass spectrometry, Gb3, globotriaosylceramide, Genz-123346, N-[(1R,2R)-1-(2,3-dihydro-1,4-benzodioxin-6-yl)-1-hydroxy-3-pyrrolidin-1-ylpropan-2-yl]nonanamide, GLUT1, glucose transporter 1, GSL, glycosphingolipid, GSL I B4, *Griffonia simplicifolia* lectin I isolectin B4, HPTLC, high-performance thin layer chromatography, HR-MS, high-resolution mass spectrometry, HUS, hemolytic uremic syndrome, MALDI-TOF, matrix-assisted laser desorption/ionization–time of flight, NMR, nuclear magnetic resonance, PNGase F, peptide-N-glycosidase F, RBC, red blood cell, SapD, human saposin D, SPR, surface plasmon resonance, STEC, Shiga toxin-producing *Escherichia coli*, Stx, Shiga toxin, Stx1B, Shiga toxin 1 B subunit, Stx2B, Shiga toxin 2 B subunit, UHPLC, ultrahigh-performance chromatography

## Abstract

The human Gb3/CD77 synthase, encoded by the *A4GALT* gene, is an unusually promiscuous glycosyltransferase. It synthesizes the Galα1→4Gal linkage on two different glycosphingolipids (GSLs), producing globotriaosylceramide (Gb3, CD77, P^k^) and the P1 antigen. Gb3 is the major receptor for Shiga toxins (Stxs) produced by enterohemorrhagic *Escherichia coli*. A single amino acid substitution (p.Q211E) ramps up the enzyme’s promiscuity, rendering it able to attach Gal both to another Gal residue and to GalNAc, giving rise to NOR1 and NOR2 GSLs. Human Gb3/CD77 synthase was long believed to transfer Gal only to GSL acceptors, therefore its GSL products were, by default, considered the only human Stx receptors. Here, using soluble, recombinant human Gb3/CD77 synthase and p.Q211E mutein, we demonstrate that both enzymes can synthesize the P1 glycotope (terminal Galα1→4Galβ1→4GlcNAc-R) on a complex type N-glycan and a synthetic N-glycoprotein (saposin D). Moreover, by transfection of CHO-Lec2 cells with vectors encoding human Gb3/CD77 synthase and its p.Q211E mutein, we demonstrate that both enzymes produce P1 glycotopes on N-glycoproteins, with the mutein exhibiting elevated activity. These P1-terminated N-glycoproteins are recognized by Stx1 but not Stx2 B subunits. Finally, cytotoxicity assays show that Stx1 can use P1 N-glycoproteins produced in CHO-Lec2 cells as functional receptors. We conclude that Stx1 can recognize and use P1 N-glycoproteins in addition to its canonical GSL receptors to enter and kill the cells, while Stx2 can use GSLs only. Collectively, these results may have important implications for our understanding of the Shiga toxin pathology.

Human Gb3/CD77 synthase (α1,4-galactosyltransferase, P1/P^k^ synthase; EC 2.4.1.228, UDP-galactose: β-D-galactosyl-β1-R 4-α-D-galactosyltransferase), encoded by the *A4GALT* gene, is a prominent enzyme in glycosphingolipid (GSL) biosynthesis (Fig. 1). It initiates the globo series GSL pathway by synthesizing the globotriaosylceramide (Gb3, Gb3Cer, CD77, P^k^ antigen, ceramide trihexoside, Galα1→4Galβ1→4Glc-Cer) from lactosylceramide (LacCer). Additionally, it generates the P1 antigen (nLc5, Galα1→4Galβ1→4GlcNAcβ1→3Galβ1→4Glc-Cer) from paragloboside (nLc4) in the neolacto series. Moreover, Gb3/CD77 synthase with a p.Q211E substitution acts further down the globo series sequentially with Gb4 synthase forming NOR1 and NOR2, both of which are determinants of the rare NOR blood group antigen, displaying a terminal Galα1→4GalNAc disaccharide (1, 2). All GSL products of human Gb3/CD77 synthase belong to the P1PK histo-blood group system (ISBT No. 003), with Gb3 referred to as the P^k^ antigen (3). The presence or absence of the P1 antigen on red blood cells (RBCs) determines the P_1_ (P1-positive) or P_2_ (P1-negative) blood group, respectively (4).

**Figure 1:**
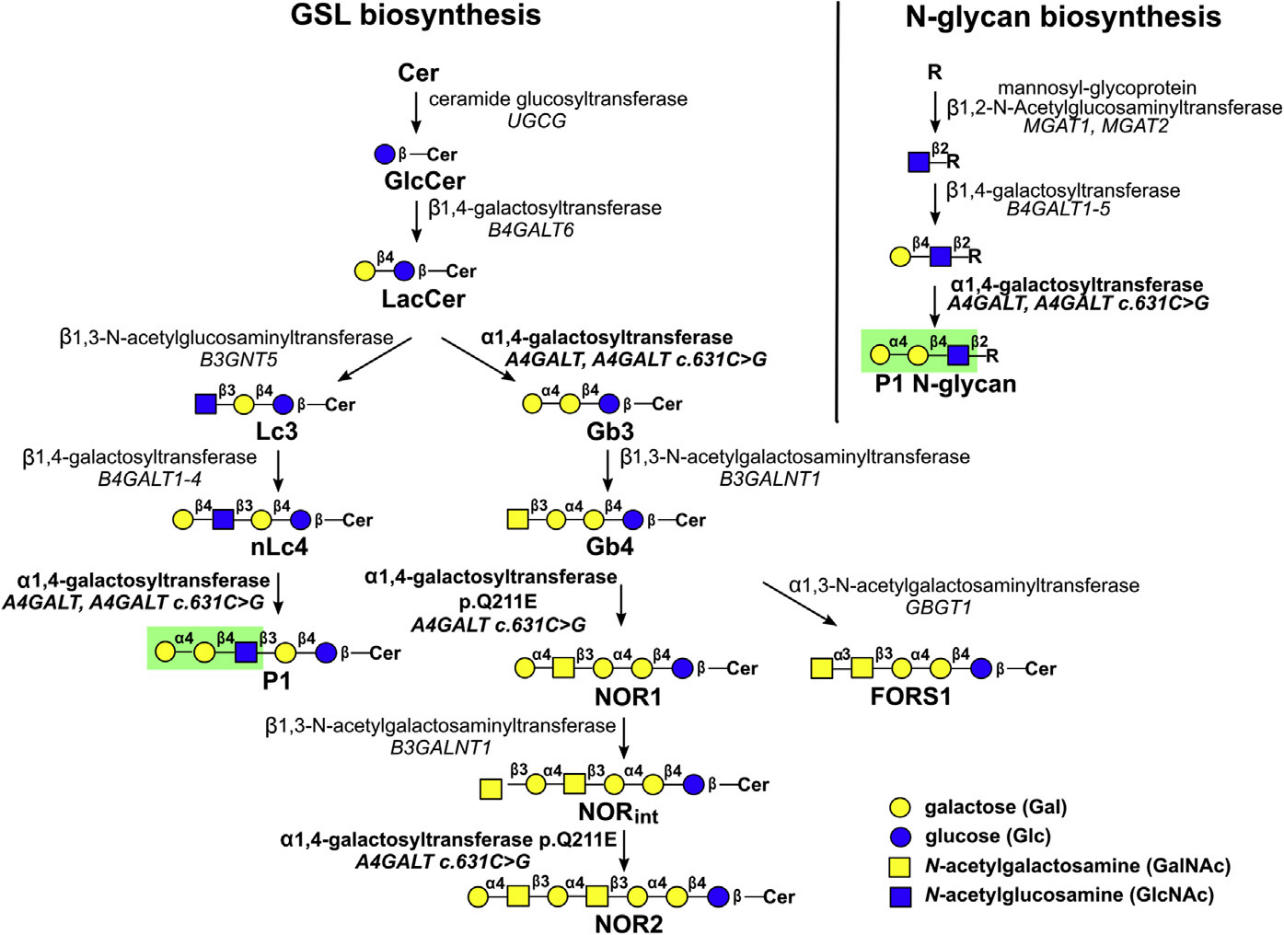
**Scheme of GSL biosynthesis and elongation of the N-glycan branch.** P1 glycotope presented on green background. Cer, ceramide; R, core N-glycan pentasaccharide.

Gb3 is one of the major neutral GSLs of human RBCs and lymphocytes, kidney, heart, lung, smooth muscle, and epithelium of gastrointestinal tract (5). Elevated levels of Gb3 have been reported in carcinomas, *i.e.*, colorectal (6), gastric (7) and ovarian cancer, where a decrease of Gb3/CD77 synthase expression and accordingly Gb3 was found to be associated with epithelial-to-mesenchymal transition (8). Accumulation of Gb3 is the hallmark of Fabry disease, caused by α-galactosidase deficiency (9). P1 expression seems to be limited to the erythroid lineage (5) and outside of RBCs, it has only been detected on ovarian cancer cells, where it was designated a cancer-associated antigen (10).

Gb3 and P1 can serve as receptors for pathogen adhesins and toxins (11, 12). Gb3 has long been established as the main receptor for Shiga toxins (Stxs), which are virulence factors produced by foodborne pathogens *Shigella dysenteriae* of serotype 1 and Shiga toxin-producing *Escherichia coli* (STEC) responsible for considerable morbidity (13, 14). Every year STEC cause an estimated 2.8 million of acute illnesses worldwide (15). Infections by STEC lead to hemorrhagic colitis, which can often progress into hemolytic uremic syndrome (HUS), a severe complication characterized by thrombocytopenia, anemia, and acute kidney failure (16). Ingested STEC secrete Stxs, which then translocate through the intestinal mucosa and reach the bloodstream, where they bind to circulating leukocytes, platelets, and RBCs (17, 18, 19, 20, 21). Stxs promote their activation and shedding of proinflammatory and prothrombotic microvesicles, which also serve as toxin carriers (19, 20, 22, 23).

Stxs produced by STEC fall into two types: Stx1, which is identical to the toxin secreted by *S. dysenteriae* of serotype 1, and more genetically distinct Stx2. Stxs display AB_5_ structure with one catalytic A domain and a pentamer of B subunits, which interact with Gb3 (5, 24). After binding to Gb3 and internalization, Stxs traverse to the Golgi, then retrogradely to the ER, where they release the catalytically active fragment of the A domain, which then translocates to the cytosol. There, the catalytic A domain fragment directly inhibits protein synthesis, induces proinflammatory cytokine expression, and activates signaling cascades leading to apoptosis (24, 25).

So far, human Gb3/CD77 synthase has been considered a strictly GSL-specific enzyme, unlike several other mammalian glycosyltransferases, which can use both GSLs and glycoproteins as acceptors. For example, human A and B transferases synthesize A and B blood group antigens on N- and O-glycans as well as on GSLs (26). In addition, at least five human β-galactosyltrasferases, three α1,3-fucosyltransferases, human and mouse sialyltransferases use glycoproteins and GSLs as acceptor substrates (27).

Previously we provided the first biochemical evidence that human Gb3/CD77 synthase is responsible for the production of both Gb3 and P1 antigen, while the same enzyme with p.Q211E substitution additionally synthesizes the rare NOR antigen (2). Here, we present evidence that human Gb3/CD77 synthase activity is not limited to GSL acceptors, but extends to complex type N-glycans and N-glycoproteins, where it can produce a terminal P1 antigen glycotope (Galα1→4Galβ1→4GlcNAc-R). Moreover, we demonstrate that glycoproteins carrying the P1 glycotope may function as receptors for Stx1, but not Stx2.

## Results

### Soluble recombinant Gb3/CD77 synthase activity toward N-glycan acceptors *in vitro*

**Figure 2:**
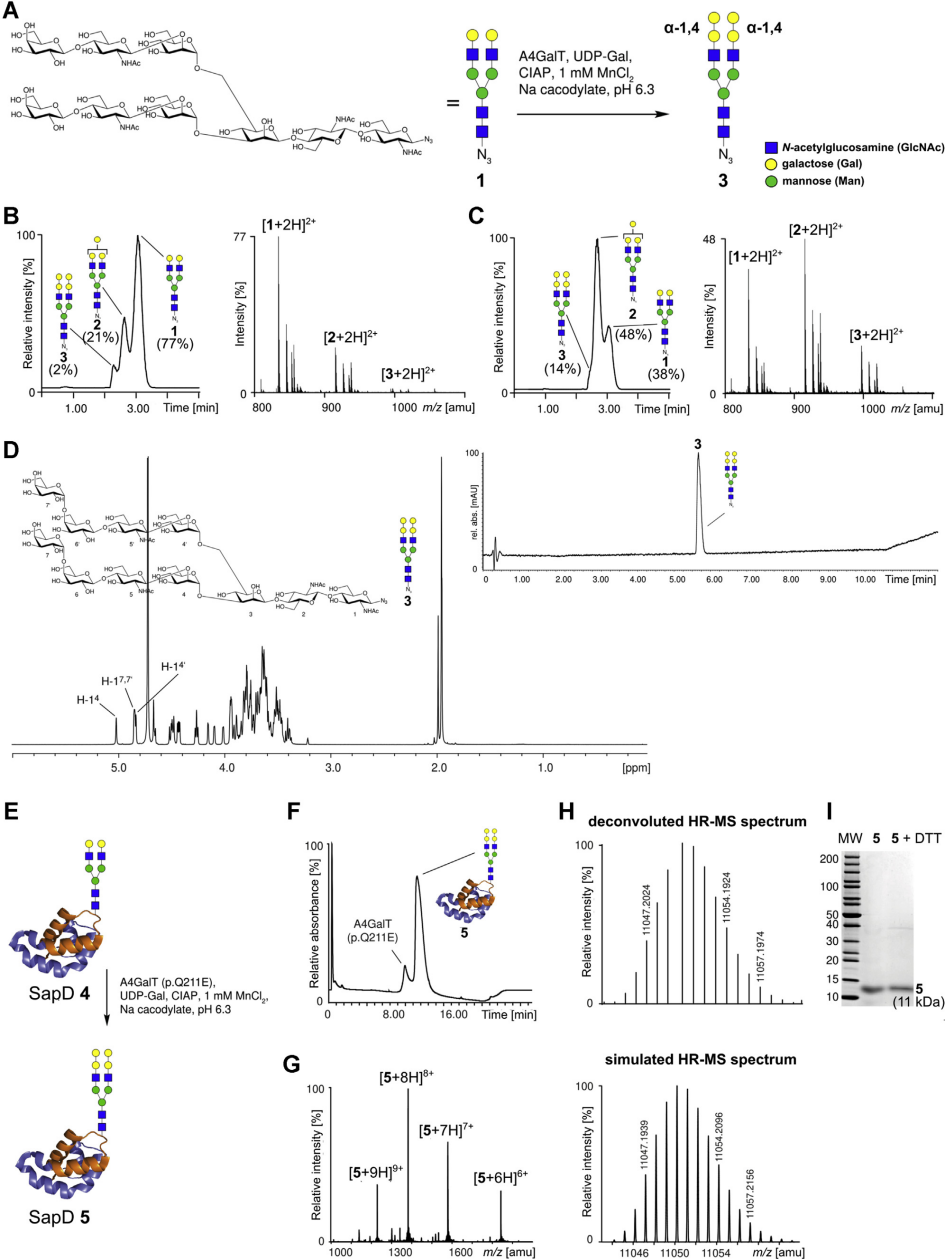
**Analysis of the conversion of N-glycan acceptors by recombinant Gb3/CD77 synthase (A4GalT) and p.Q211E mutein (A4GalT p.Q211E) *in vitro*. *A*,** structure of nonasaccharide azide **1** and reaction conditions for incubations with A4GalT. ***B***, total ion count (TIC) from HR-LC-MS of **1** incubated for 24 h with A4GalT, summed up mass spectrum from TIC. ***C***, total ion count (TIC) from HR-LC-MS of **1** incubated for 24 h with A4GalT p.Q211E, summed up mass spectrum from TIC. ***D***, ^1^H-NMR (500 MHz, D_2_O) and UHPLC trace of purified undecasaccharide azide **3**. ***E*,** cartoon of SapD glycoform **4** and reaction conditions for incubations with A4GalT p.Q211E. ***F*,** UHPLC trace of the synthesis of SapD glycoform **5**. ***G*,** HR-MS of purified SapD glycoform **5**. ***H*,** deconvoluted and simulated HR-MS spectrum of the [M+9H]^9+^ peak of **5**. ***I*,** SDS-PAGE of purified SapD glycoform **5** under nonreducing (central lane) and reducing conditions (right lane). CIAP, calf intestinal alkaline phosphatase; SDS-PAGE, sodium dodecyl sulphate - polyacrylamide gel electrophoresi.

The catalytic domain of human Gb3/CD77 synthase and the p.Q211E mutein were expressed recombinantly in insect cells with an N-terminal 6xHis Tag. The reactivity of both glycosyltransferases toward N-glycans was tested in 50 mM cacodylate buffer (pH 6.3) containing UDP-Gal, Mn^2+^ and alkaline phosphatase (28). As an acceptor substrate was selected nonasaccharide azide **1** with two terminal lactosamine units (29) (Fig. 2*A*). Reverse phase high-performance liquid chromatography-mass spectrometry analysis after incubation with the p.Q211E mutein showed new glycans with higher masses corresponding to one and two additional galactose units. Since the LC-peaks of the N-glycans were not well resolved and conversion was incomplete, the concentration of acceptor **1** was varied (1–10 mM). Only the reaction with 10 mM **1** and p.Q211E mutein gave nearly complete conversion after 6 days of incubation (data not shown). Subsequently, the activities of Gb3/CD77 synthase and the p.Q211E mutein were compared. LC-MS analysis of both reactions after 24 h showed mainly acceptor **1** (77%) for the Gb3/CD77 synthase (Fig. 2*B*), whereas for the p.Q211E mutein only 38% of **1** remained (Fig. 2*C*). For the decasaccharide intermediates **2,** the conversions were 21% *versus* 48%, and for the undecasaccharide product, **3** 2% *versus* 14%, respectively (Fig. 2, *B* and *C*). To determine the linkage of the transferred galactose units, a preparative reaction was carried out, lasting 7 days, followed by purification of **3** by gel filtration and ultrahigh-performance chromatography (UHPLC). The product **3** was obtained in 84% yield and was characterized by LC-MS and NMR (Fig. 2*D*). In the 500 MHz NMR spectrum, the anomeric signals (H-1) of the two newly transferred galactoses H-1^7^ and H-1^7'^ overlap with H-1^4^ but show a small coupling constant indicating their α-configuration. This is further ascertained by the large ^1^*J*_CH_ coupling constants for H-1^7^, C-1^7^ and H-1^7'^, C-1^7'^ of 170.6 Hz. Both α-galactoses 7 and 7' are attached to O-4 of the β-galactoses 6 and 6' resulting in the typical downfield shift of the ring carbon after glycosylation (C-4^6^ and C-4^6'^ at 78.2 ppm).

**Figure 3:**
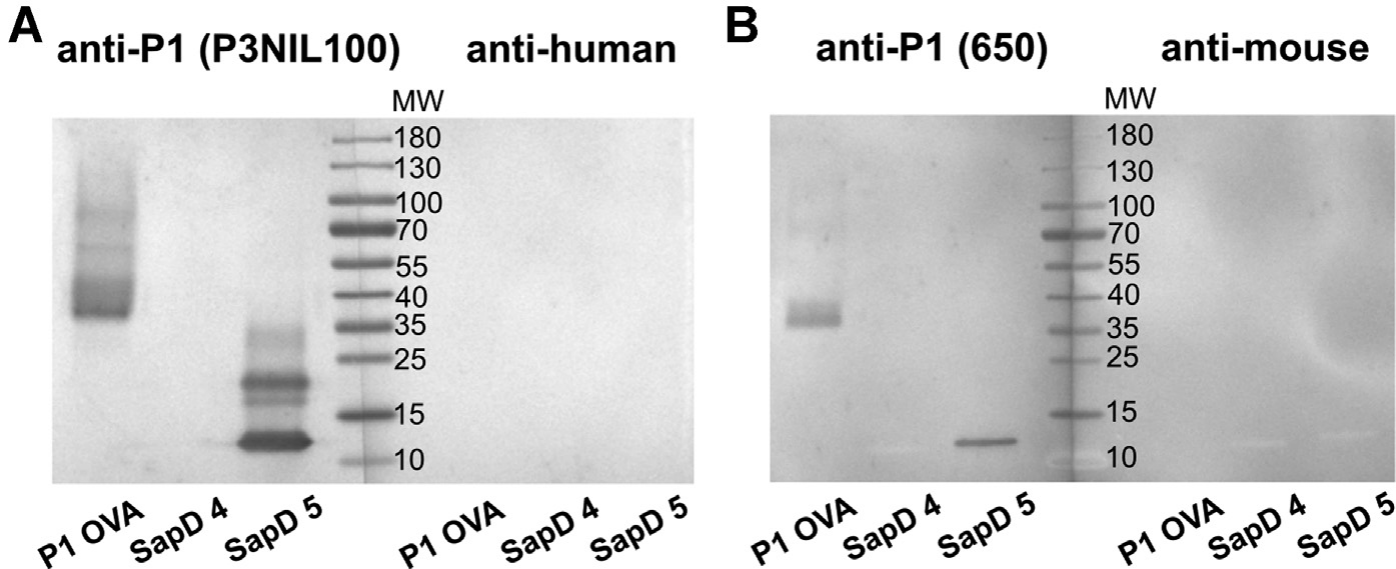
**Western blotting analysis of SapD 4 and SapD 5 using anti-P1 antibodies: P3NIL100 (*A*), 650 (*B*).** The bands of higher molecular weight than 11 kDa are presumably artifacts caused by boiling the sample. P1 OVA, pigeon egg ovalbumin containing P1 glycotope used as the positive control.

We next investigated the possibility of α-galactosylating N-glycopeptides. Since both Gb3/CD77 synthase and its p.Q211E mutein showed the ability to galactosylate the nonasaccharide azide **1**, but p.Q211E mutein appeared to act faster, we decided to use the mutein in the next approach. A synthetic human saposin D (SapD) 1-35 aa. glycopeptide hydrazide, bearing the biantennary N-glycan part of **1**, was tested as a substrate and gave full conversion with the p.Q211E mutein even at an acceptor concentration of 1 mM (data not shown). A synthetic glycoform of SapD (**4**) (30) also showed full conversion after incubation of 1 mM **4** with the p.Q211E mutein in the presence of UDP-Gal for 7 days (Fig. 2, *E* and *F*). The reaction was purified by UHPLC and gave the new SapD glycoform **5** in a yield of 78%. HR-MS analysis of purified **5** (Fig. 2*G*) revealed the addition of two hexose units and virtual identity of the deconvoluted (Fig. 2*H*) and the simulated spectrum. The sodium dodecyl sulphate - polyacrylamide gel electrophoresis showed the purity of the synthetic glycoprotein **5** (Fig. 2*I*) and the presence of terminal Galα1→4Galβ1→4GlcNAc structures on the N-glycan termini of SapD **5** was confirmed by western blotting using anti-P1 monoclonal antibodies (p3NIL100 and 650, specificities detailed in Table S1), which bound to SapD **5** in contrast to SapD **4** (Fig. 3). Artifactual bands of higher molecular weight than 11 kDa are visible only in SapD **5** sample after blotting with P3NIL100 antibody because of its high sensitivity (Fig. 3*A*).

### Recombinant Gb3/CD77 synthase activity toward N-glycan acceptors *in vivo*

**Figure 4:**
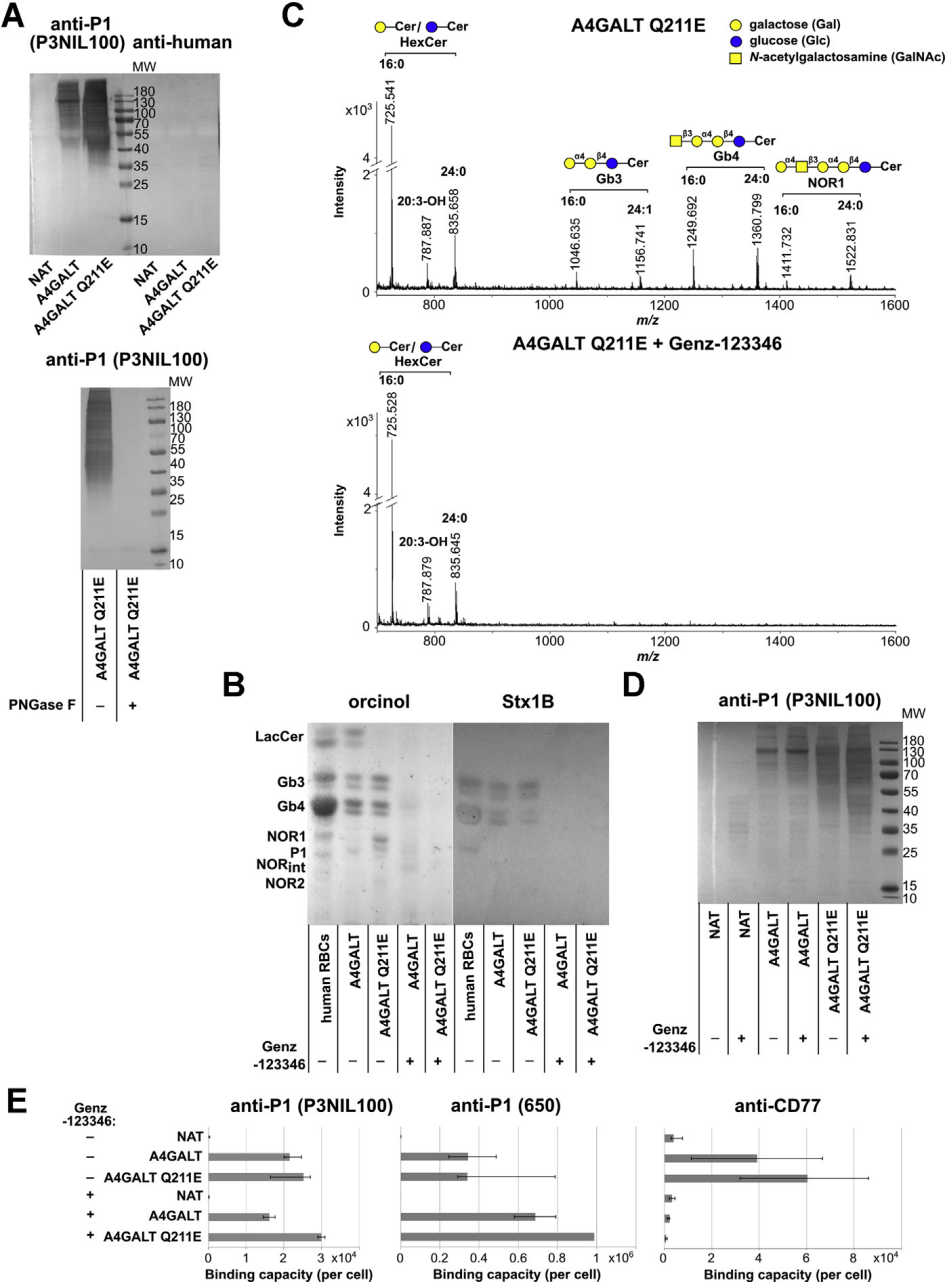
**Effects of human Gb3/CD77 synthase expression on CHO-Lec2 glycoproteins and GSLs. *A*,** western blotting analysis of CHO-Lec2 cell lysates stained with anti-P1 (P3NIL100) antibody and analysis of PNGase F-treated and untreated lysates of CHO-Lec2 A4GALT Q211E cells. ***B*,** HPTLC analysis of neutral glycosphingolipids purified from CHO-Lec2 cells stained with orcinol and Stx1B. ***C*,** MALDI-TOF analysis of neutral glycosphingolipids isolated from CHO-Lec2 A4GALT Q211E cells, untreated or cultured in the presence of Genz-123346. ***D*,** western blotting analysis of Genz-123346-treated and untreated CHO-Lec2 cell lysates. ***E*,** quantitative flow cytometry analysis of anti-P1 (P3NIL100), anti-P1 (650) and anti-CD77 binding capacity per CHO-Lec2 cell (displayed as median from at least three replicates;error bars represent interquartile ranges). A4GALT, CHO-Lec2 A4GALT; A4GALT Q211E, CHO-Lec2 A4GALT Q211E; human RBCs, human RBCs of *P*^*1NOR*^*P*^*1*^ genotype used as the positive control; NAT, untransfected CHO-Lec2.

CHO-Lec2 cells were transduced with a lentiviral vector encoding the full-length human Gb3/CD77 synthase (CHO-Lec2 A4GALT) or its p.Q211E mutein (CHO-Lec2 A4GALT Q211E). The anti-P1 antibody (P3NIL100) detected Galα1→4Galβ1→4GlcNAc structures on several glycoproteins in CHO-Lec2 A4GALT and CHO-Lec2 A4GALT Q211E cell lysates (Fig. 4*A*). After PNGase F treatment, no binding was detected suggesting that these structures were present only on N-linked, but not on O-linked glycan chains. There was no binding to the untransduced cell lysate. GSL I B4 lectin did not bind to CHO-Lec2 A4GALT and CHO-Lec2 A4GALT Q211E cell lysates, indicating a lack of Galα1→3Gal structures (Fig. S1*A*). Similarly, anti-NOR antibody (nor118) did not detect any glycoproteins in CHO-Lec2 cell lysates, suggesting that they do not contain Galα1→4GalNAc structures (Fig. S1*B*).

To answer the question whether Gb3/CD77 synthase produces P1 glycotope on GSLs only, or on GSLs and glycoproteins, CHO-Lec2 cells were cultured in the presence of Genz-123346, which is a glucosylceramide synthase inhibitor (31). After 2 weeks of culture in the Genz-123346-containing medium, no GSLs were detected in high-performance thin layer chromatography (HPTLC) analysis of CHO-Lec2 A4GALT and CHO-Lec2 A4GALT Q211E (Fig. 4*B*), and matrix-assisted laser desorption/ionization–time of flight (MALDI-TOF) only detected a hexosylceramide (which probably was galactosylceramide) (Fig. 4*C*). Significantly, the binding of anti-P1 antibody (P3NIL100) to the lysates of Genz-123346-treated cells in western blotting was increased in comparison with the untreated cell lysates (Fig. 4*D*).

The binding of anti-P1 (P3NIL100 and 650) and anti-CD77 to CHO-Lec2 cells was evaluated by quantitative flow cytometry (Fig. 4*E*). Both anti-P1 antibodies recognize Galα1→4Gal structures not only on GSLs but also on glycoproteins, in contrast to anti-CD77, which binds solely Gb3 (specificities detailed in Table S1). The binding levels of anti-P1 (P3NIL100) to CHO-Lec2 A4GALT and CHO-Lec2 A4GALT Q211E treated with Genz-123346 and untreated cells were similar, in contrast to anti-P1 (650), the binding of which was increased. Thus, CHO-Lec2 A4GALT and CHO-Lec2 A4GALT Q211E cells treated with Genz-123346 produce at least that much of P1 glycoproteins as untreated cells. In contrast, anti-CD77 antibody did not bind to CHO-Lec2 A4GALT and CHO-Lec2 A4GALT Q211E treated with Genz-123346, suggesting that these cells did not produce Gb3.

**Figure 5:**
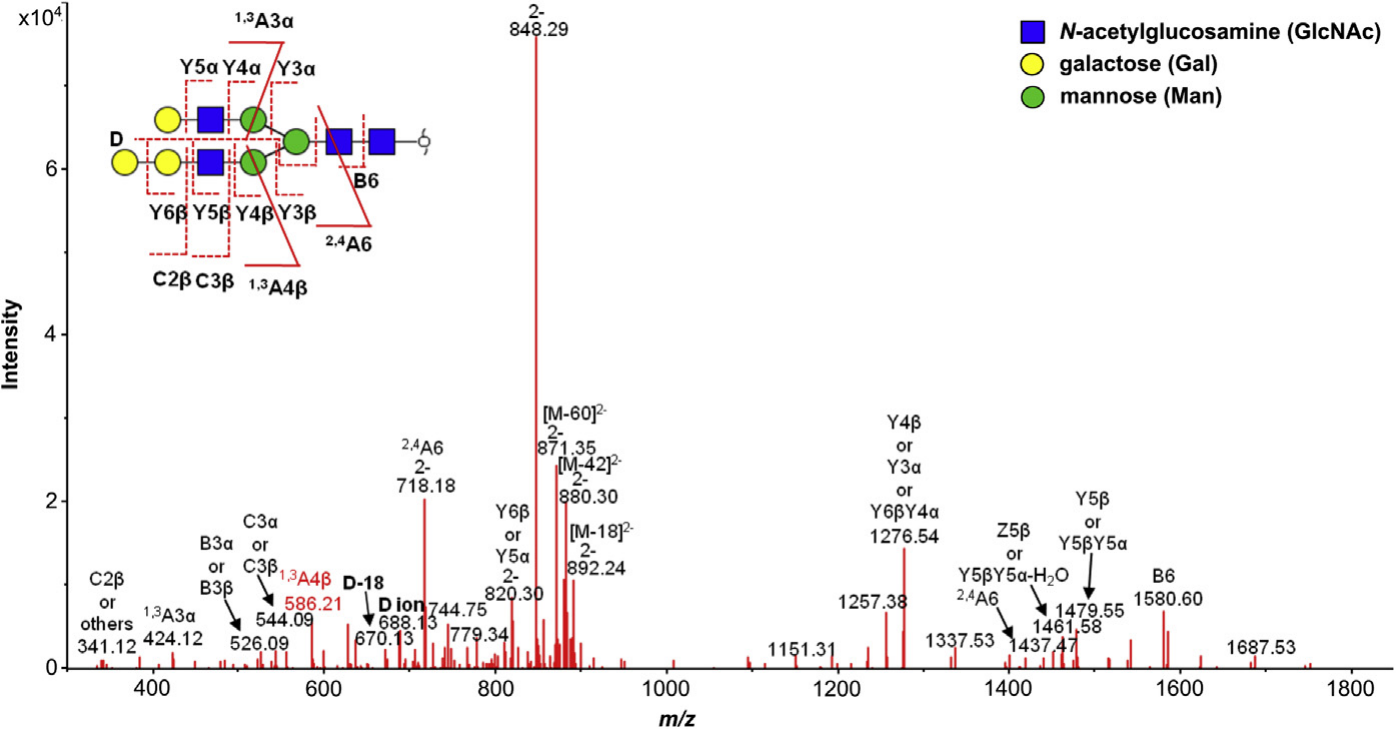
**Tandem MS spectrum of an N-glycan at *m/z* 901.34**^**2**−^**of the CHO-Lec2 A4GALT Q211E analyzed by PGC nano-LC-ESI-MS/MS in negative ion mode.**

To further characterize the changes in N-glycans of CHO-Lec2 cells induced by Gb3/CD7 synthase and p.Q211E mutein expression, N-glycans were enzymatically released from total cellular protein preparations, reduced, and analyzed by porous graphitized carbon LC-MS. CHO-Lec2 cells showed expression of mainly oligomannosidic N-glycans. The major complex-type glycans were identified as a biantennary, fully galactosylated N-glycans with and without core fucose (H5N4F0-1). CHO-Lec2 A4GALT and CHO-Lec2 A4GALT Q211E showed largely similar N-glycan profiles, but exhibited an additional decasaccharide N-glycan species at *m/z* 901.33 [M-2H]^2−^ (Fig. S2*A*) at a relative abundance of 0.77% (standard deviation 0.10%) and 1.07% (standard deviation 0.03%), respectively (Fig. S2*B*). Of note, this decasaccharide was not detected in the untransfected CHO-Lec2 cells. Notably, tandem mass spectrometry of this species revealed a ^1,3^A4 fragment ion at *m/z* 586.21 indicating the addition of a hexose to one of the antennae (Fig. 5). The D ion at *m/z* 688.13 and D-18 ion at *m/z* 670.13 indicated this modification to present on the three-linked antenna rather than on the six-linked antenna. No other N-glycans with an additional antennary hexose were detected when screening the tandem mass spectra for the diagnostic ion of *m/z* 586.21. These additional hexose-modified N-glycans may well occur, albeit below the detection limit of the LC-MS method. Together, these data indicate the addition of a hexose to the three-linked antenna of a biantennary complex type N-glycan in CHO-Lec2 cells upon expression of Gb3/CD77 synthase, which points to the presence of an antennary Galα1→4Galβ1→4GlcNAc motif.

### P1 glycoproteins are recognized by Stx1B, but not Stx2B

**Figure 6:**
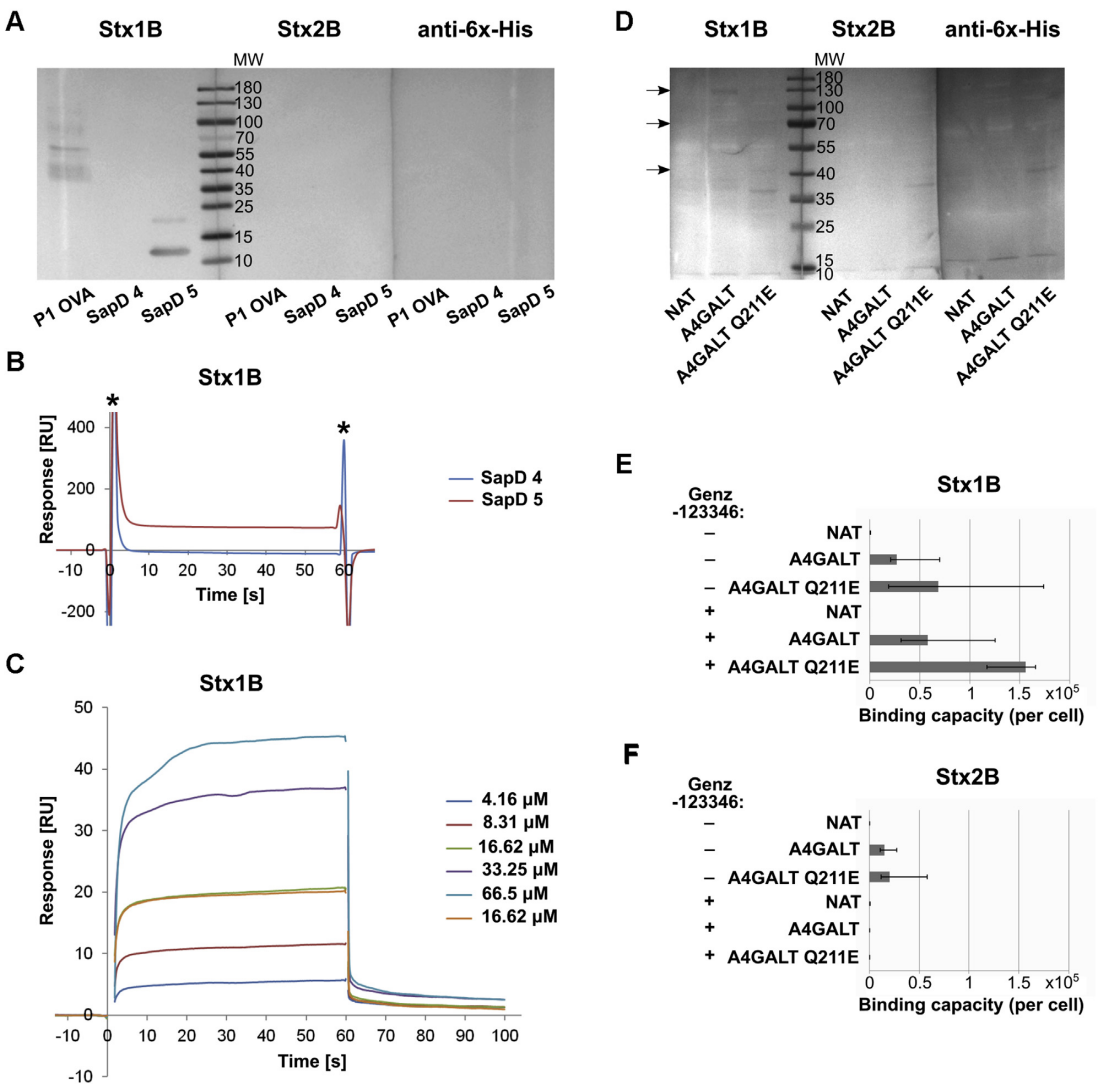
**Interactions of Stx1B and Stx2B with glycoproteins. *A*,** western blotting analysis of SapD **4** and SapD **5** with Stx1B and Stx2B. The band of molecular weight at approximately 22 kDa is presumed to be a SapD **5** artefact. P1 OVA, pigeon egg ovalbumin containing P1 glycotope, was used as the positive control. ***B*,** SPR binding analysis of Stx1B to SapD **4** and SapD **5**. ***C*,** SPR affinity analysis of Stx1B interaction with SapD **5**. ∗, spikes resulted from the difference between composition of the running buffer and sample bufer. ***D*,** western blotting analysis of CHO-Lec2 cell lysates stained with Stx1B and Stx2B. ***E*,** quantitative flow cytometry analysis of Stx1B binding capacity per CHO-Lec2 cell (displayed as median from at least three replicates; error bars represent interquartile ranges). ***F*,** quantitative flow cytometry analysis of Stx2B binding capacity per CHO-Lec2 cell (displayed as median from at least three replicates; error bars represent interquartile ranges). A4GALT, CHO-Lec2 A4GALT; A4GALT Q211E, CHO-Lec2 A4GALT Q211EP1 OVA, pigeon egg ovalbumin containing P1 glycotope used as the positive control; NAT, untransfected CHO-Lec2.

To determine whether Shiga toxin 1 or 2 can bind to glycoproteins containing P1 glycotope, we performed a western blotting analysis of SapD **4** and SapD **5** using recombinant Shiga toxin 1 and 2 B subunits (Stx1B and Stx2B). Stx1B recognized only SapD **5**, while Stx2B did not bind any of the SapD glycoforms (Fig. 6*A*). The binding of Stx1B to SapD **4** and SapD **5** immobilized on the sensor chip CM5 was evaluated by SPR analysis. Stx1B bound to SapD **5**, whereas no interaction was observed for SapD **4** (Fig. 6*B*). A single affinity analysis for SapD **5** interactions with different concentrations of Stx1B (4.16, 8.31, 16.62, 33.25, 66.5 μM) reported calculated dissociation constant, *K*_D_ = 3.31 × 10^−5^ M (Fig. 6*C*).

Moreover, western blotting analysis of CHO-Lec2 A4GALT and CHO-Lec2 A4GALT Q211E cell lysates revealed that Stx1B binds several glycoproteins (Fig. 6*D*), whereas Stx2B did not detect any proteins. Next, we evaluated Stx1B and Stx2B binding to CHO-Lec2 A4GALT and CHO-Lec2 A4GALT Q211E cells using flow cytometry (Fig. 6, *E* and *F*). CHO-Lec2 A4GALT Q211E cells showed increased Stx1B and Stx2B binding capacities in comparison with CHO-Lec2 A4GALT. After Genz-123346 treatment, Stx1B binding to CHO-Lec2 A4GALT and CHO-Lec2 A4GALT Q211E cells was approximately two times higher than to the untreated cells (Fig. 6*E*), whereas Stx2B binding was abolished (Fig. 6*F*).

### The p.Q211E mutein produces more Gb3 and P1 glycoproteins than Gb3/CD77 synthase

The results of western blotting and flow cytometry analysis of CHO-Lec2 cells suggested that the p.Q211E mutein exhibits a stronger activity toward glycoprotein substrates than Gb3/CD77 synthase (Figs. 4, *A* and *E* and S1*B*). Moreover, flow cytometry analysis of CHO-Lec2 cells with anti-CD77 antibody and HPTLC analysis using orcinol staining and overlay with Stx1B and anti-P1 (650) antibody revealed that CHO-Lec2 A4GALT Q211E cells produced more Gb3 than CHO-Lec2 A4GALT (Figs. 4, *B* and *E* and S3*A*). Quantitative analysis of the *A4GALT* transcript revealed that CHO-Lec2 A4GALT Q211E cells express approximately ten times less *A4GALT* mRNA than CHO-Lec2 A4GALT (Table 1). Likewise, anti-A4GALT antibody (5C7) detected significantly less p.Q211E protein in CHO-Lec2 A4GALT Q211E cell lysates in comparison with Gb3/CD77 synthase protein in CHO-Lec2 A4GALT (Fig. S3*B*). Thus, the elevated levels of p.Q211E mutein products did not result from upregulation of the *A4GALT* transcript, nor did they result from an increase in enzyme production.

**Table 1:** Quantitative analysis of *A4GALT* transcripts in CHO-Lec2 cells

Cells	ΔCt	ΔΔCt	RQ
NAT	15.7123	0	1
A4GALT	−0.1155	−15.8278	58,163.7
A4GALT Q211E	3.2034	−12.5089	5828.3
NAT + Genz-123346	16.4905	0	1
A4GALT + Genz-123346	−0.6479	−17.1384	144269.5
A4GALT Q211E + Genz-123346′	3.5537	−12.9369	7841.2

RQ, relative quantity.

**Figure 7:**
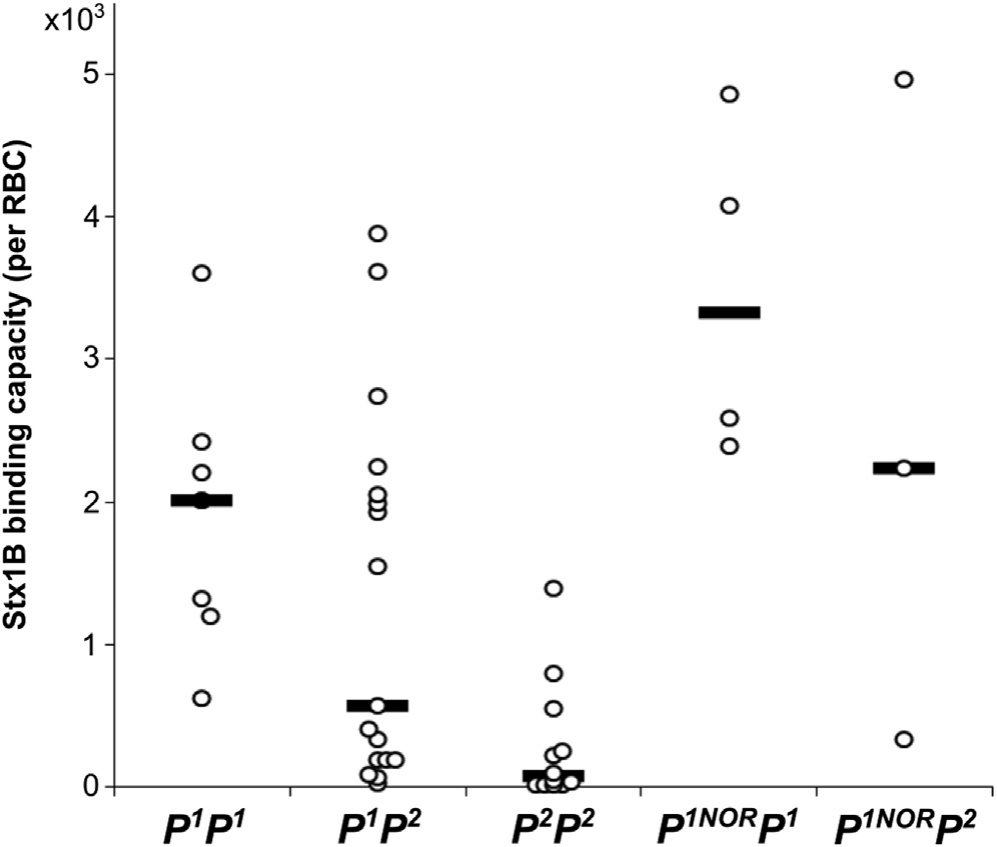
**Quantitative flow cytometry analysis of Stx1B binding capacity per RBC in individuals of different genotypes** (the lines are medians).

We sought to determine if the higher activity of the p.Q211E mutein observed in CHO-Lec2 cells was unique to that cell line or representative also for cells of human origin. Therefore, we analyzed RBCs of different *A4GALT* genotypes using quantitative flow cytometry. Table 2 summarizes relationships between phenotypes in terms of P1PK blood group system, *A4GALT* genotypes, and the presence or absence of Gb3/CD77 synthase GSL products on the RBC surface. *P*^*1NOR*^*P*^*1*^ and *P*^*1NOR*^*P*^*2*^ genotypes showed elevated Stx1B binding compared with *P*^*1*^*P*^*1*^and *P*^*1*^*P*^*2*^, respectively (Fig. 7).

**Table 2:** Phenotypes of P1PK histo-blood group system and *A4GALT* genotypes

Phenotype	Genotype	Antigens
P_1_	*P*^*1*^*P*^*1*^	P1, P^k^
	*P*^*1*^*P*^*2*^	
P_2_	*P*^*2*^*P*^*2*^	P^k^
P	ψψ	-
P_1_NOR	*P*^*1NOR*^*P*^*1*^	P1, P^k^, NOR1, NOR2, NOR_int_
	*P*^*1NOR*^*P*^*2*^	

P^k^, Gb3; ψ, allele not encoding any functional protein.

### P1 glycoproteins serve as functional receptors for Stx1, but not Stx2

**Figure 8:**
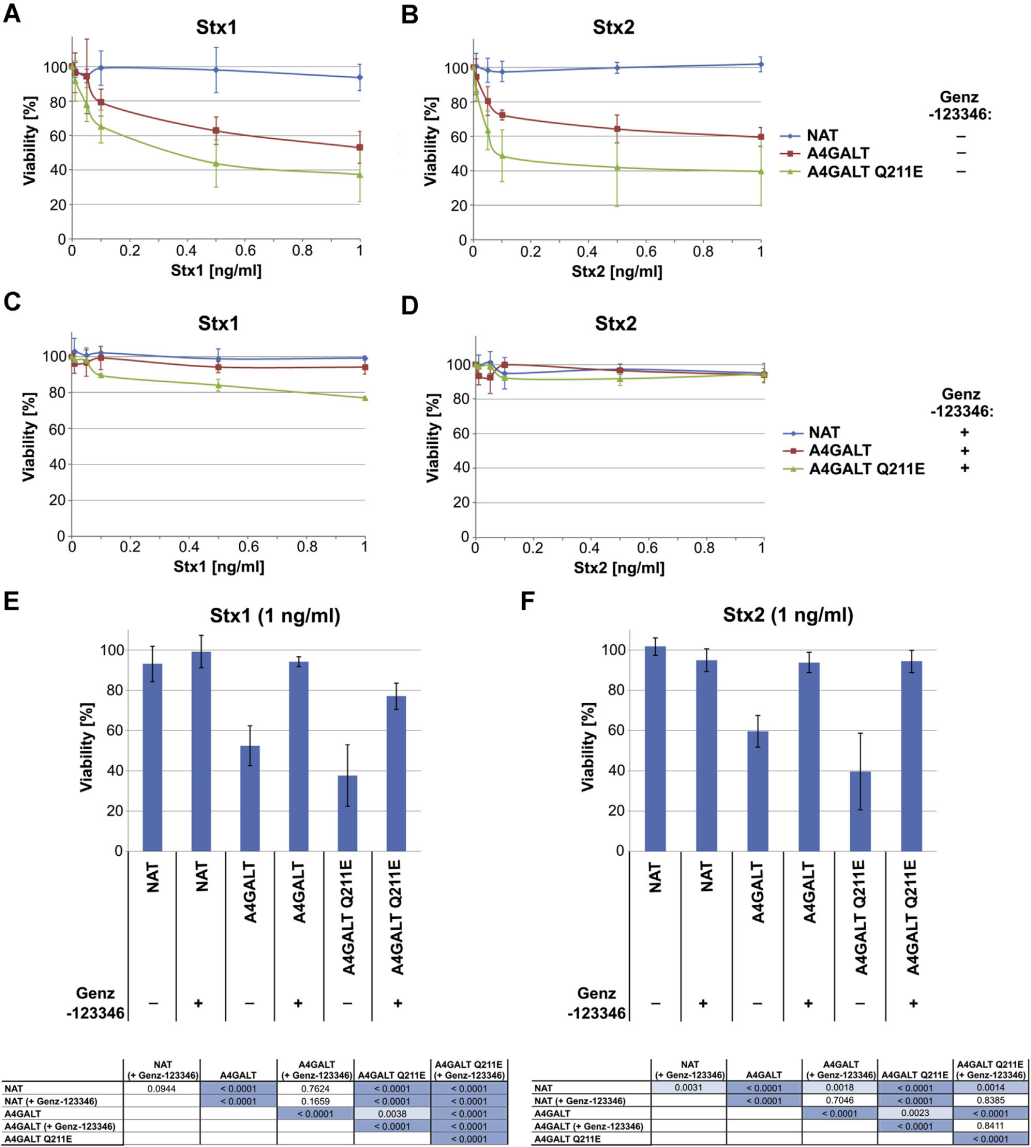
**Stx cytotoxicity analysis.** Viability of CHO-Lec2 cells after exposure to Stx1 **(*A*)** or Stx2 **(*B*)** (at least three independent experiments were conducted, each with three technical replicates; error bars are standard deviations). Viability of CHO-Lec2 cells cultured in the presence of Genz-123346 treated with Stx1 **(*C*)** or Stx2 **(*D*)** (at least three independent experiments were conducted, each with three technical replicates; error bars are standard deviations). CHO-Lec2 viability cultured in the presence or absence of Genz-123346 after 20 h of 1 ng/ml Stx1 **(E)** or Stx2 **(F)** treatment (mean; error bars are standard deviations). Tables contain *p* values of the *t* test with Holm–Bonferroni correction (*p* < 0.05; *p* < 0.01; *p* < 0.001). A4GALT, CHO-Lec2 A4GALT; A4GALT Q211E, CHO-Lec2 A4GALT Q211E; NAT, untransfected CHO-Lec2.

To examine whether Stx1 and Stx2 can use P1 glycoproteins as functional receptors, we performed cytotoxicity assays on CHO-Lec2 A4GALT and CHO-Lec2 A4GALT Q211E cells. Untransduced CHO-Lec2 cells were not sensitive to Stx1 and Stx2 and served as the negative control. We found that CHO-Lec2 A4GALT Q211E cells were more sensitive to Stx1 (Fig. 8, *A* and *E*) and Stx2 (Fig. 8, *B* and *F*) in comparison with CHO-Lec2 A4GALT. While this may be due to elevated Gb3 synthesis in CHO-Lec2 A4GALT Q211E cells, the increased sensitivity to Stx1 may be also caused by an increased amount of P1 glycoproteins. After Genz-123346 treatment, CHO-Lec2 A4GALT Q211E cells were still susceptible to Stx1 (but to a lesser extent than the untreated cells) (Fig. 8, *C* and *E*) in contrast to CHO-Lec2 A4GALT cells, which displayed a lower level of P1 glycoproteins. Strikingly, the cells cultured in the presence of Genz-123346 were refractory to the Stx2 treatment (both CHO-Lec2 A4GALT and CHO-Lec2 A4GALT Q211E) (Fig. 8, *D* and *F*), which suggests that P1 glycoproteins serve as functional receptors for Stx1, but not for Stx2.

## Discussion

The P1PK histo-blood group system continues to perplex. The conundrum of its genetic and biochemical background was covered in depth in reviews and original reports by us and other groups (4, 32, 33, 34, 35). One view that remained largely unchallenged for over 20 years was that the human Gb3/CD77 synthase (α1,4-galactosyltransferase, P1/P^k^ synthase; EC 2.4.1.228) could only use GSLs as acceptors to produce Galα1→4Gal-terminated glycans (Gb3 and P1). The presence of P1 glycotope on human glycoproteins was first suggested by (36) and tested by (37), who reported that P1-antiserum recognized band 4.5 (GLUT1) and, to a lesser extent, other erythrocyte membrane proteins. However, a subsequent study contradicted those results by showing a complete depletion of P1 determinants from erythrocyte membrane glycoproteins upon treatment with *n*-butanol (38). Since then, there was a general agreement that GSLs are the only carriers of the P1 glycotope. Recently (39), suggested that some human erythrocytes of P_1_ phenotype display P1 not just on GSLs, but also on glycoproteins, and implicated GLUT1 and Band 3 as likely major carriers of the P1 glycotope. However, the evidence was limited to western blotting results.

Human Gb3/CD77 synthase is an unusual glycosyltransferase insofar that it creates one (Gb3) or two (Gb3 and P1) GSL products depending on its gene expression level (32). The enzyme becomes even more promiscuous upon substitution of one amino acid residue, p.Q211E, which renders it able to synthesize four different GSLs (Gb3, P1, NOR1, and NOR2) (1).

Here, we evaluated the hypothesis that human Gb3/CD77 synthase can also produce P1 glycotopes on glycoproteins. We used the soluble catalytic domain of human Gb3/CD77 synthase and the p.Q211E mutein obtained from a baculovirus-insect cell expression system (2) and evaluated their activity toward the biantennary N-glycan acceptor **1** (29). We found that both Gb3/CD77 synthase and its p.Q211E mutein readily transfer galactose residues to both antennae of the acceptor; however, the p.Q211E mutein provides a faster reaction. In a follow-up experiment, we tested the activity of p.Q211E toward **4**, a synthetic glycoform of human saposin D (30). This small lysosomal glycoprotein contains one N-glycosylation site at Asn22 and was synthesized as a pure glycoform carrying a complex nonasaccharide N-glycan with both antennae terminating in Galβ1→4GlcNAc (**4**). Thus, it served as a good model glycoprotein to verify the ability of the Gb3/CD77 synthase to also accept glycoproteins as substrates. We found that the enzyme can readily attach galactose residues to both antennae of the N-glycan of SapD **4** *in vitro*. The presence of P1 glycotopes on SapD **5** was confirmed by western blotting.

These results were corroborated by cell line studies, for which we selected CHO-Lec2 cells because they do not express an endogenous Gb3/CD77 synthase. Additionally, they are deficient in CMP-sialic acid transporter, so they are incapable of sialylation, which may compete with α-galactosylation during the biosynthesis of N-glycans (40, 41, 42). We found that CHO-Lec2 cells expressing human Gb3/CD77 synthase or its p.Q211E mutein produced P1 glycoproteins, with the mutein being the more efficient enzyme. Removal of N-glycans rendered the glycoproteins undetectable by the anti-P1 antibody, indicating that P1 glycotopes were present only on N-, but not O-linked glycans. To ensure the absence of GSLs as potential confounders in protein lysates, we used Genz-123346, which is an inhibitor of glucosylceramide synthase. Inhibition of glucosylceramide synthase blocks the synthesis of globo and neolacto series GSLs, including the substrates for Gb3/CD77 synthase. Indeed, the cells exposed to Genz-123346 did not produce any GSL substrates or products of the enzyme, while expressing more P1 glycoproteins. Collectively, these results strongly indicate that human Gb3/CD77 synthase and the p.Q211E mutein can synthesize P1 glycotope on N-glycans of glycoproteins. This was confirmed by direct mass spectrometric analysis of the released N-glycans, which indicated the presence of the P1 glycotope on a biantennary N-glycan upon expression of Gb3/CD77 synthase or p.Q211E mutein.

For years, it was taken for granted that human Gb3/CD77 synthase could act exclusively on GSLs, so there was no logical prerequisite to look for human proteins recognized by Stx. Hitherto, an article published by (43) is the sole report conjecturing the existence of putative mammalian proteins recognized by Stx1 and Stx2 in Gb3 deficient Vero cells. However, the P1 glycotope has been found on avian and amphibian glycoproteins (44). Pigeon egg white ovomucoid expressing P1 on N-glycans is recognized by Stx1 and can be used in affinity chromatography for purification of the toxin (42, 45). A recombinant mucin-type fusion protein PSGL-1/mIgG_2b_ produced in CHO-K1 cells, which were cotransfected with vectors encoding the pigeon homolog of human Gb3/CD77 synthase and human core 2 β1,6-*N*-acetylglucosaminyltransferase, expressed P1 glycotopes on O-glycans (46). That glycoprotein, along with human serum albumin conjugated with the Gb3 glycan (Galα1→4Galβ1→4Glc3-atom spacer-NH-HSA), was recognized by Stx1, but not Stx2 as probed by western blotting and SPR analysis.

In this study, we found that the Stx1, but not Stx2 B subunit, can recognize a synthetic model glycoprotein carrying P1 glycotope as well as unidentified glycoproteins on mammalian cells overexpressing human Gb3/CD77 synthase or its hyperactive mutein (p.Q211E) and on RBCs of P_1_ phenotype. We propose that Stx1B recognizes both GSLs and glycoproteins, whereas Stx2B binds to GSLs only.

Using CHO-Lec2 cells, we found that p.Q211E mutein produces more Gb3 and P1 glycoproteins in comparison with Gb3/CD77 synthase, despite its lower expression at the mRNA and protein level. The P1 GSL was not synthesized at all despite *A4GALT* overexpression (hence Gb3 is the sole GSL product of Gb3/CD77 synthase detected in CHO-Lec2, whereas p.Q211E mutein synthesizes Gb3 and the NOR antigen). However, we previously demonstrated that on the surface of human RBCs of *P*^*1NOR*^*P*^*1*^ and *P*^*1NOR*^*P*^*2*^ genotypes (with one allele encoding p.Q211E mutein), antibodies detected less Gb3 and P1 antigen in comparison with *P*^*1*^*P*^*1*^ and *P*^*1*^*P*^*2*^ genotypes, respectively (32). To further examine this issue, we also tested Stx1B binding to these RBCs. Strikingly, RBCs of *P*^*1NOR*^*P*^*1*^ and *P*^*1NOR*^*P*^*2*^ genotypes showed increased binding in comparison with *P*^*1*^*P*^*1*^ and *P*^*1*^*P*^*2*^, respectively. The discrepancy is probably caused by methodological and molecular differences between the anti-P1 and Stx1B binding to their targets. Prior to anti-P1 antibody assays, RBCs are treated with papain to allow efficient recognition of GSL epitopes, which otherwise may be cloaked by membrane proteins. Papain is a nonselective protease, so it may inadvertently remove P1 glycoproteins along with the others. In contrast, papain treatment is not necessary for the analysis with the use of Stx1B and may even markedly decrease the binding (Fig. S4). Stx binding subunits and antibodies have different affinities for Gb3 and P1 GSLs and vastly differ in size, so these receptors may be less accessible to antibodies (24, 47). Taken together, we presume that the p.Q211E mutein indeed produces the P1 GSL less efficiently (hence the decreased anti-P1 binding to protease-treated RBCs), but it exhibits a higher activity toward glycoprotein acceptors in comparison with Gb3/CD77 synthase (hence the increased Stx1B binding).

While Gb3 significance as the Stx receptor is generally accepted, the existence of other receptors is still a matter of debate. Gb3 is further elongated into globoside (Gb4, GalNAcβ1→3Galα1→4Galβ1→4Glc-Cer) and subsequently into Forssman antigen (FORS1, GalNAcα1→3GalNAcβ1→3Galα1→4Galβ1→4Glc-Cer), both of which are considered additional receptors for Stx2e variant responsible for pig edema disease (48, 49). Gb4 has been reported as a low-affinity receptor for a few Stx variants, but the evidence is inconclusive and requires further clarification (50, 51, 52, 53).

Due to the long-standing notion that only GSLs carry the Galα1→4Gal disaccharide in humans, glycoproteins were never considered as potential Stx receptors. Since we found that human Gb3/CD77 synthase (and p.Q211E mutein to an even greater extent) is able to produce P1 glycoproteins and they are recognized by Stx1B, we examined whether they can serve as functional Stx receptors, capable of mediating cytotoxicity. In cytotoxicity assays, cells expressing p.Q211E mutein exhibited a higher susceptibility to Stx1 and Stx2. Results of assays with untreated cells were very similar for both Stx1 and 2; however, the cells with abolished globo and neolacto series GSL synthesis became refractory to Stx2. Strikingly, while neither Stx1 nor Stx2 was able to kill GSL-deficient cells that overexpressed Gb3/CD77 synthase, cells overexpressing the p.Q211E mutein were still partially susceptible to Stx1 despite the depletion of GSLs. Thus, P1 glycoproteins present on the cell surface are sufficient to induce Stx1 cytotoxicity, likely by acting as functional, internalizing receptors. We presume that Gb3/CD77 synthase produced too little P1 glycotopes on glycoproteins to make the cells vulnerable to Stx1. These results are consistent with the Stx1B and Stx2B binding experiments. Taken together, Stx1 can recognize and use P1 glycoproteins to enter and kill the cell, while Stx2 can use GSL receptors only.

The ability to synthesize carbohydrate chains of both glycoproteins and GSLs by one enzyme is not uncommon. One example is ABO histo-blood group system transferases, all of which synthesize A and B blood group antigens on different N-, O-, and Cer-linked glycans depending on the cell type, *e.g.*, on RBCs and endothelium predominantly on type 2 chain (Galβ1→4GlcNAc-R), whereas in gastrointestinal epithelium on type 1 chain (Galβ1→3GlcNAc-R) (5). It is estimated that 80% of ABH determinants on RBCs are present on glycoproteins and 20% on GSLs (54). Human β1,3-galactosyltransferases 1, 2, and 5 produce type 1 chain, and β1,4-galactosyltransferase 1 and 5 type 2 chain structures on glycoproteins as well as on GSLs (55, 56, 57, 58). Human and mouse β1,3*-N*-acetylglucosaminyltransferase 5 uses GSL and glycoprotein acceptors (59). Human α1,3-fucosyltransferase 4, 6, and 9 synthesize Le^x^ antigen on N-glycans, O-glycans, and GSLs (60). Human and mouse α2,8-sialyltransferase 3, human and mouse ST6 *N*-acetylgalactosaminide α2,6-sialyltransferase 3, human α2,3-sialyltransferase 6, mouse α2,3-sialyltransferase 1 and 2 act on glycoproteins and GSLs (61, 62, 63, 64, 65). Our results place Gb3/CD77 synthase in the same category as the abovementioned glycosyltransferases. On the contrary, synthesis of Galα1→3Gal structures on mammalian GSLs and glycoproteins is accomplished by distinct enzymes: isoglobotriaosylceramide synthase (*A3GALT2*) and glycoprotein α1,3-galactosyltransferase 1 (*GGTA1*), respectively (66).

Our findings also prompt the question of whether Gb3/CD77 synthase favors GSL substrates over glycoproteins or it shows no clear preference. We estimate that the rate of transfer to glycoprotein acceptors in comparison with GSLs measured *in vivo* is less than 5%. On the other hand, the rate *in vitro* (*i.e.*, against purified N-glycans or purified proteins) may well approach 100%, as we showed with saposin D. Unfortunately, we cannot reliably infer substrate preferences from analyses of enzyme activity with synthetic or model structures (nonasaccharide azide or saposin D and synthetic glycoconjugates carrying GSL-type glycans) (2). We presume that the difference in Gb3/CD77 synthase activity toward GSLs and glycoproteins *in vivo* is most probably caused by the morphology of the Golgi compartment, where glycosyltransferases may form complexes (including heterodimers), which act as conveyor belts for their substrates. Formation of such glycosyltransferase complexes has been well documented but often underappreciated, as many researchers tend to study glycosyltransferases individually (67). For example, human β1,4-galactosyltransferase 1 (*B4GALT1*) and β-galactoside α2,6-sialyltransferase (*ST6GAL1*) form heterodimers in the Golgi using lateral interactions by highly charged surface domains (68). Using Bimolecular Fluorescence Complementation Assay (BiFC), we found before that the human Gb3/CD77 synthase forms heterodimers with β1,4-galactosyltransferase 6 (*B4GALT6*), which is specific toward GSLs (unpublished results). Formation of such heterodimers may commit enzymes to individual pathways and, in the case of Gb3/CD77 synthase, prevent it from making complexes with glycosyltransferases that synthesize N-glycans, such as β1,4-galactosyltransferase 1 (*B4GALT1*). As a result, Gb3/CD77 synthase may prefer GSLs not necessarily because of its acceptor specificity, but because of its ability to form complexes with other GSL-synthesizing enzymes, such as β1,4-galactosyltransferase 6 (*B4GALT6*). Further comprehensive studies will be necessary to elucidate that complex phenomenon.

A recently published article showed that glycoproteins carrying the P1 glycotope synthesized by the pigeon homolog of human Gb3/CD77 synthase may serve as functional receptors for Stx1 (69). The authors knocked out the genes encoding glucosylceramide and galactosylceramide synthases in HeLa cells, and used these cells to overexpress a pigeon homolog of human Gb3/CD77 synthase. The authors also expressed the human Gb3/CD77 synthase in these cells and showed that they can produce P1 glycoproteins, albeit at levels too low to induce Stx1 cytotoxicity. A disruption of the gene encoding α1,3-mannosyl-glycoprotein 2-β-*N*-acetylglucosaminyltransferase (but not glycoprotein-*N*-acetylgalactosamine 3-β-galactosyltransferase 1) caused a decrease in Stx1B binding to HeLa cells expressing the pigeon homolog of human Gb3/CD77 synthase. Thus, it may be argued that P1 glycotopes on glycoproteins are synthesized in HeLa cells only on N-glycans, which is consistent with our experiments involving removal of N-glycans from CHO-Lec2 glycoproteins. The unmodified HeLa cells produce Gb3, but not P1 glycoproteins. Interestingly, when Morimoto *et al*. expressed the pigeon homolog of human Gb3/CD77 synthase in unmodified HeLa and obtained cells producing both Gb3 and P1 glycoproteins, these were less sensitive to Stx1 than unmodified cells (69). The authors suggest that P1 glycoproteins served as decoy receptors for the toxin, but it should be noted that HeLa cells expressing the pigeon homolog produced less Gb3 than unmodified cells, so it could be that the P1 glycoproteins were not able to compensate for the loss of Gb3, the GSL being a more potent receptor. Our study design prevented potential misinterpretations of cytotoxicity patterns. Notably, in our cytotoxicity assays, the sensitivity of cells producing both Gb3 and P1 glycoproteins was comparable for both Stx1 and Stx2. Since P1 glycoproteins are recognized by Stx1, but not Stx2, we could expect a higher sensitivity for Stx1. We hypothesize that the functionality of P1 glycotopes on glycoproteins as receptors for Stx1 depends on their proportion to Gb3 molecules on the cell surface.

The results of this study have several broader implications. The current understanding of Stx pathology in humans is based on the long-standing view that the toxins can use only GSLs to enter and kill the cells. A large body of data has been collected on how the toxins engage the receptors, invaginate the cell membrane, and escape degradation once inside the cell to make their way to the ER and damage the ribosomes (24, 70). Several complex mechanistic requirements have to be met for productive engagement of the GSL receptors, which include a proper composition of the ceramide moiety and membrane microenvironment. A mismatch may lead to a failure to enter the cell or escape trafficking to lysosomes and degradation. In light of that, it is somewhat surprising that glycoproteins, representing a vastly different type of molecules, can substitute for or perhaps even complement the role of GSLs. This opens up a new avenue for research on the Shiga toxin pathology. For the time being, the difference between Stx1 and 2 receptor preferences that we found here may be related to the difference in virulence between Stx1 and Stx2-producing STEC strains, the latter of which are more virulent in humans, but pinpointing that link will require further investigation. Finally, our findings militate against the potential to use soluble P1-carrying protein decoys as Shiga toxin blocking agents in therapy for STEC infection, at least in the case of Stx1-producing strains. Uptake of toxin-decoy complexes could potentially lead to activation of ribotoxic stress response in antigen presenting cells and exacerbate the dysregulation of immune response seen in severe STEC infections, should the toxin be able to escape degradation. Overall, our findings may indicate the need to shift focus from designing efficient Galα1→4Gal-decorated binders to inventing agents that would irreversibly sequestrate the toxins.

## Experimental procedures

### Antibodies

The human anti-P1 (clone P3NIL100, recognizing only P1 antigen), mouse anti-P1 (clone 650, recognizing Gb3 and P1 antigen), mouse anti-CD77-FITC (clone 5B5, recognizing Gb3), anti-6x-His Tag antibody (clone HIS.H8), and biotinylated goat anti-mouse IgG/A/M (H/L) antibodies were purchased from Immucor Inc, Ce-Immundiagnostika, BioLegend, Thermo Fischer Scientific, and Bio-Rad Laboratories respectively. The goat anti-human polyvalent immunoglobulins biotinylated antibody and alkaline phosphatase-ExtrAvidin were obtained from Sigma-Aldrich. The mouse monoclonal anti-NOR (clone nor118, recognizing NOR antigen) (71) and anti-A4GALT (clone 5C7, recognizing Gb3/CD77 synthase) antibody were used as a diluted culture supernatant.

Anti-A4GALT (clone 5C7) mouse monoclonal antibody was obtained by Dr. Arkadiusz Miazek, as described in (72).

### Shiga toxins

The recombinant Stx1a and Stx2bB His-tagged subunits (referred to as Stx1B and Stx2B), used in western blotting and flow cytometry analysis, were obtained as described (73).

Stx1B used in the SPR analysis was expressed and purified as described below. Briefly, *stx1B* gene was amplified on genomic DNA of *E. coli* O157:H7 strain EDL933 (ATCC 700927) using primers StxBNStart (5’ GGATCCCATATGAAAAAAACATTATTAATAGCTGCATC 3’), StxXhoHind (5’ AAGCTTCTCGAGACGAAAAATAACTTCGCTGAATCC 3’) and cloned into NdeI-HindIII sites of pET21b(+) plasmid (Novagen). The pET21b-stx1B construct was introduced into *E. coli* BL21(DE3)pLysS (Novagen), which were cultured in LB medium containing 100 μg/ml ampicillin, 34 μg/ml chloramphenicol, and 3% ethanol in an orbital shaker (37 °C, 220 rpm) until OD_600_ reached 0.8. Then *stx1B* gene expression was induced with 1 mM IPTG and the culture was incubated (30 °C, 220 rpm) until the OD_600_ reached ∼2. The bacteria were centrifuged (4800*g*, 4 °C, 10 min) and the pellet was dispersed in 25 ml of buffer Stx-A (50 mM Tris-HCl, 150 mM NaCl, 10 mM 2-mercaptoethanol, pH 7.8). Lysozyme (Roth) was added to the concentration of 1 mg/ml, followed by a 30 min incubation at room temperature. Cell disintegration was performed in Cell Disruptor One Shot (Constant Systems Ltd) at the pressure of 20 kpsi and imidazole was added to the lysate to the concentration of 40 mM. The lysate was applied to the equilibrated HisTrap HP 1 ml prepacked column (GE Healthcare), followed by washing with ten column volumes of buffer Stx-A with 40 mM imidazole. The protein was eluted with 500 μl fractions of buffer Stx-A with 250 mM imidazole.

The Stx1 (Verotoxin 1, from *E. coli* O157) and Stx2 (Verotoxin 2, from *E. coli* O157) holotoxins for cytotoxicity assays were purchased from Sigma-Aldrich (USA).

### Expression of recombinant catalytic domain of human Gb3/CD77 synthase in insect cells

Recombinant soluble catalytic domain of human Gb3/CD77 synthase and its p.Q211E mutein were expressed and purified as described (2). Briefly, a fragment of the *A4GALT* gene spanning nucleotides 130–1059 of the ORF with C or G at position 631 respectively was cloned in-frame with c-myc and 6x-His tags into pGEM-T Easy Vector (Promega). The constructs were used to obtain high-titer suspensions of recombinant baculoviruses (ordered from GenScript) harboring the cloned *A4GALT*-c-myc-6x-His fragment downstream to the gp67 secretion signal sequence. Suspension cultures of Sf9 cells, maintained in serum-free medium (Sf900 II SFM, Life Technologies) at 27 °C and 110 rpm, were infected with one of the recombinant baculoviruses at a multiplicity of infection (MOI) of five virus particles per Sf9 cell. Forty-eight hours after infection, cells were discarded and the supernatant was subjected to purification on Ni-NTA resin (Ni- NTA Agarose, Qiagen). Protein concentration was determined using Bradford assay.

### Evaluation of Gb3/CD77 synthase activity toward N-glycan acceptors *in vitro*

The nonasaccharide azide **1** was prepared from egg yolk as described previously (29). Three milligram (1.8 μmol, 1 eq) of **1** was dissolved in 150 μl of buffer (50 mM sodium cacodylate, pH 6.3) containing bovine serum albumin (1.2 mg/ml), alkaline phosphatase (12 mU/μl, E.C. 3.1.3.1, calf intestine), MnCl_2_ (1.2 mM), and UDP-Gal (5.4 μmol, 3 eq). Recombinant soluble Gb3/CD77 synthase or p.Q211E mutein (30 μl, 233 μg) was added to the mixture. After 7 days of incubation at 30°C, the mixture was purified by size-exclusion chromatography (HiLoad 16/600 Superdex 30 pg, 0.1 M NH_4_HCO_3_, flow: 0.6 ml/min). After lyophilization, the crude product **3** was further purified by RP-HPLC (YMC Hydrosphere C18, 150 × 10 mm, 5 μm, 0–3% MeCN/H_2_O containing 0.1% formic acid; Pharmacia Äkta Purifier 100). Electrospray ionization-time-of-flight mass spectra of the reaction product were recorded on a Micromass LCT instrument coupled to a Waters H-Class UHPLC System. High-resolution ESI mass spectra were recorded on a Thermo Q Exactive Orbitrap mass spetrometer coupled to a Dionex Ultimate 3000 UHPLC System. NMR spectra were recorded with a Bruker Advance III HD 500 instrument. The undecasaccharide **3** was characterized and assigned by ^1^H and ^13^C resonances from a couple of 1D and 2D NMR experiments (JMOD, HH-COSY, HH-TOCSY, HSQC, HSQC-TOCSY, NOESY). The spectra were calibrated using [D4]-Methanol (δ(1H) = 3.31 ppm, δ(13C) = 49.00 ppm). Coupling constants are reported in Hz and the resonances are assigned according to Figure 2*D*.

^1^H-NMR (500 MHz, D_2_O, [D_4_]-methanol as internal standard): δ = 5.12 (d, 1H, *J*_1,2_ < 1 Hz, H-1^4^α), 4.96 to 4.92 (m, 3H, H-1^7^α, H-1^7'^α, H-1^4'^α), 4.77 to 4.74 (m, 2H, H-1^3^β, H-1^1^β), 4.63 to 4.56 (m, 3H, H-1^2^β, H-1^5^β, H-1^5'^β), 4.55 to 4.50 (m, 2H, H-1^6^β, H-1^6'^β), 4.40 to 4.32 (m, 2H, H-5^7^, H-5^7'^), 4.25 (dd, 1H, *J*_1,2_, *J*_2,3_ < 1 Hz, H-2^3^), 4.19 (dd, 1H, *J*_1,2_, *J*_2,3_ < 1 Hz, H-2^4^), 4.11 (dd, 1H, *J*_1,2_, *J*_2,3_ < 1 Hz, H-2^4'^), 4.06 to 3.53 (m, 59H, H-4^6^, H-4^6'^, H-4^7^, H-4^7'^, H-6a^3^, H-6a^2^, H-6a^6^, H-6a^6'^, H-3^6^, H-3^6'^, H-6a^4^, H-6a^4'^,H-3^7^, H-3^7'^, H-3^4^, H-3^4'^, H-6a^1^, H-6b^2^, H-2^7^, H-2^7'^, H-6b^6^, H-6b^6'^, H-2^2^, H-5^6^, H-5^6'^, H-3^2^, H-6b^3^, H-3^3^, H-4^2^, H-4^1^, H-4^3^, H-5^1^, H-6b^1^, H-2^1^, H-2^5^, H-5^4^, H-5^4'^, H-2^5'^, H-6a,b^7^, H-6a,b^7'^, H-6a,b^5^, H-6a,b^5'^, H-3^5^, H-3^5'^, H-4^5^, H-4^5'^, H-6b^4^, H-6b^4'^, H-5^2^, H-3^1^, H-5^3^, H-5^5^, H-5^5'^, H-2^6^, H-2^6'^), 3.53 to 3.45 (m, 2H, H-4^4^, H-4^4'^), 2.10 to 2.02 (m, 12H, NAc). ^13^C-NMR (125 MHz, D_2_O, [D_4_]-methanol as internal standard): δ = 175.8, 175.7, 175.6 (C=O, NAc), 103.9 (C-1^6^β, ^1^*J*_C-1,H-1_ = 162.8 Hz), 103.9 (C-1^6'^β, ^1^*J*_C-1,H-1_ = 161.7 Hz), 102.3 (C-1^2^β, ^1^*J*_C-1,H-1_ = 162.5 Hz), 101.4 (C-1^3^β, ^1^*J*_C-1,H-1_ = 160.7 Hz), 101.3 (C-1^7^α, ^1^*J*_C-1,H-1_ = 170.6 Hz), 101.3 (C-1^7^α, ^1^*J*_C-1,H-1_ = 170.6 Hz), 100.5 (C-1^4^α, ^1^*J*_C-1,H-1_ = 171.3 Hz), 100.4 (C-1^5^β, ^1^*J*_C-1,H-1_ = 162.8 Hz), 100.4 (C-1^5'^β, ^1^*J*_C-1,H-1_ = 164.5 Hz), 98.0 (C-1^4'^α, ^1^*J*_C-1,H-1_ = 170.6 Hz), 89.5 (C-1^1^β, ^1^*J*_C-1,H-1_ = 158.7 Hz), 81.4 (C-3^3^), 80.4 (C-4^2^), 79.8 (C-4^5^), 79.7 (C-4^5'^), 79.6 (C-4^1^), 78.2 (C-4^6^), 78.2 (C-4^6'^), 77.4 (C-51), 77.3 (C-2^4^), 77.2 (C-2^4^´), 76.4 (C-5^6^), 76.4 (C-5^6'^), 75.7 (C-5^5^), 75.7 (C-5^5'^), 75.4 (C-5^2^), 75.4 (C-5^3^), 74.5 (C-5^4^), 73.8 (C-5^4'^), 73.2 (C-3^5^), 73.2 (C-3^5'^), 73.1 (C-3^6^), 73.1 (C-3^6'^), 73.0 (C-3^1^), 73.0 (C-3^2^), 71.9 (C-2^6^), 71.9 (C-2^6'^), 71.8 (C-5^7^), 71.8 (C-5^7'^), 71.2 (C-2^3^), 70.4 (C-3^4^), 70.4 (C-3^4'^), 70.1 (C-3^7^), 70.1 (C-3^7'^), 69.9 (C-4^7^), 69.9 (C-4^7'^), 69.5 (C-2^7^), 69.5 (C-2^7'^), 68.3 (C-4^4^), 68.3 (C-4^4'^), 66.7 (C-6^3^), 62.7 (C-6^4^), 62.7 (C-6^4'^), 61.4 (C-6^7^), 61.4 (C-6^7'^), 61.3 (C-6^6^), 61.3 (C-6^6'^), 61.0 (C-6^1^), 61.0 (C-6^2^), 61.0 (C-6^5^), 62.6 (C-6^5'^), 56.0 (C-2^2^), 55.9 (C-2^5^), 55.9 (C-2^5'^), 55.5 (C-2^1^), 23.3, 23.2, 23.1 (NAc).

The synthetic glycoform SapD **4** (30) was used as an acceptor for the soluble catalytic domain of Gb3/CD77 synthase produced in insect cells. In total, 100 μg (9.3 nmol, 1 eq) of **4** was dissolved in 9.2 μl of buffer (50 mM sodium cacodylate, pH 6.3) containing alkaline phosphatase (10 mU/μl, E.C. 3.1.3.1, calf intestine), MnCl_2_ (1 mM), UDP-Gal (37.7 nmol, 4 eq), and p.Q211E mutein (31 μg). The reaction was incubated at 30 °C and monitored by HR-MS (ESI-MS in H_2_O, 0.1% formic acid, flow 0.1 ml/min). After 7 days, the reaction was purified by RP-HPLC (Supelco Discovery BIO Wide Pore C5, 50 × 2.1 mm, 5 μm, gradient: 20–80% MeCN/H_2_O containing 0.1% formic acid, flow: 0.5 ml/min), and fractions containing the product **5** were pooled and lyophilized (Pharmacia Äkta Purifier 100).

### Surface plasmon resonance (SPR) analysis of Stx1B binding to SapD 4 and SapD 5

Interactions of Stx1B (analyte) with SapD **4** and SapD **5** (ligands) were evaluated on Biacore T-200 (GE Healthcare), using HBS-N (GE Healthcare) as the running buffer. For the immobilization of the ligands on Series S Sensor Chip CM5, the Amine Coupling Kit (GE Healthcare) was used. Ligands (SapD **4** and **5**) were diluted in 10 mM sodium acetate buffer, pH 4.5, to the concentration of 10 μg/ml. The final immobilization levels of SapD **4** and SapD **5** were approximately 70 and 60 response units (RU), respectively. Unmodified flow cells served as reference surface. For binding analysis, 93.97 μM Stx1B was injected over the sensor surface for 1 min. For affinity analysis, Stx1B, at the following concentrations: 4.16, 8.31, 16.62 (repeated twice), 33.25, 66.5 μM, was injected for 1 min at the flow rate 50 μl/min and the dissociation was monitored for 5 min. The flow of running buffer was sufficient to regenerate the surface. All measurements were performed at 25 °C and analyzed using BIAevaluation Software 4.0 (GE Healthcare). *K*_D_ was determined by fitting to the steady-state affinity model.

### Lentiviral expression of a full-length human Gb3/CD77 synthase and p.Q211E mutein in CHO-Lec2 cell line

*A4GALT* and *A4GALT C631G* ORFs were amplified by PCR from pCAG vectors established before (1) with the following primers: forward (5’→3’) GGAATTCGATACCATGTCCAAGCCCCCCG and reverse (5’→3’) GAACGCGTTCACAAGTACATTTTCATGGCCT. PCR products were subjected to EcoRI and MluI restriction enzyme digestion and cloned into lentiviral expression vector pRRL-CMV-IRES-PURO (all vectors for lentiviral expression system were kindly provided by Prof D. Trono, École Polytechnique Fédérale de Lausanne, Switzerland). Then, vectors containing *A4GALT* or *A4GALT C631G* ORF (pRRL-CMV-A4GALT-IRES-PURO and pRRL-CMV-A4GALT.Q211E-IRES-PURO) were used for Lenti-X 293T cells (Clontech) transfection with 0.05 mg/ml polyethyleneimine (Sigma-Aldrich), as described (74). Briefly, cells at 50–60% confluency were cotransfected with 20 μg/ml of pRRL-CMV-A4GALT-IRES-PURO or pRRL-CMV-A4GALT.Q211E-IRES-PURO, 10 μg/ml of pMDL-g/p-RRE, 5 μg/ml of pRSV-REV, and 5 μg/ml pMk-VSVG vectors in serum-free medium (αMEM). The medium was changed 24 h and harvested 72 h after transfection. After centrifugation (400*g*, 5 min, 24 °C), supernatant-containing lentiviruses were concentrated 100× on Amicon Ultra-15K:100 000 (Milipore).

CHO-Lec2 cells were obtained from the American Type Culture Collection. Culture of CHO-Lec2 was maintained in DMEM/F12 (Gibco) supplemented with 10% fetal bovine serum (Gibco) at 37 °C under humidified 5% CO_2_ atmosphere. Cells were subcultivated every 3–4 days with 0.25% trypsin/1 mM EDTA. Transduction was performed by centrifugation (2460*g*, 150 min, 24 °C) of 2 × 10^4^ cells with concentrated lentiviruses, as described (74). After 24 h, cells were subjected to puromycin selection (CHO-Lec2 A4GALT 40 μg/ml and CHO-Lec2 A4GALT Q211E 15 μg/ml, respectively) to obtain stable clones.

For some experiments, CHO-Lec2 A4GALT, CHO-Lec2 A4GALT Q211E, and untransfected cells were cultured for 2 weeks in complete medium containing 3 μM *N*-[(1*R*,2*R*)-1-(2,3-dihydro-1,4-benzodioxin-6-yl)-1-hydroxy-3-pyrrolidin-1-ylpropan-2-yl]nonanamide (Genz-123346) (Merck), a glucosylceramide synthase inhibitor.

### Western blotting

The SapD glycoform **5** and P1 glycotope containing pigeon ovalbumin (P1 OVA, purified from *Columba livia* egg) were subjected to nonreducing sodium dodecyl sulphate - polyacrylamide gel electrophoresis on 4–20% Tris-Glycine gel. CHO-Lec2 cells were lysed in CelLytic M (Sigma-Aldrich) supplemented with protease inhibitor cocktail. In total, 60 μg of cell lysate per lane was subjected to nonreducing sodium dodecyl sulphate - polyacrylamide gel electrophoresis on 10% Tris-Glycine gel and transferred to nitrocellulose membrane. The membrane was blocked in 5% cold fish gelatin in TBS for 1 h, washed three times (all washing steps done with TBS), and incubated overnight with primary antibody diluted 1:100 in TBS/1% BSA. After fivefold washing, the membrane was placed for 1 h in 1:1000 biotinylated secondary antibody solution, washed, and incubated with ExtrAvidin conjugated with alkaline phosphatase (1:5000, 1 h). The bands were visualized using BCIP/NBT reaction.

PNGase F digestion of CHO-Lec2 protein lysates was performed as described (75). Briefly, 1 μl of 10% SDS and 0.7 μl 1 M DTT were added to 60 μg of cell lysate, and the samples were incubated in 60 °C for 5 min. Then they were subjected to deglycosylation with recombinant PNGase F in 50 mM sodium phosphate buffer, pH 7.5 for 2 h in 37 °C.

### Mass spectrometry analysis of CHO-Lec2 N-glycans

N-glycans of the CHO-Lec2 cells were analyzed as described previously (76). In short, cells were either harvested by trypsin treatment or alternatively scraped off, pelleted, washed, and stored until further use. Cell pellets were homogenized, and (glyco-)proteins were adsorbed to PVDF membrane, followed by enzymatic N-glycan release, reduction, and analysis by porous graphitized carbon nanoLC-MS nanoelectrospray ion trap MS/MS (AmazonSpeed ion trap; Bruker Daltonics) using a nanoBooster. Tandem mass spectra were manually assigned following the collision-induced glycan fragmentation rules for negative-mode MS summarized in a recent review article (77). Glycan tandem mass spectra including those of the doubly-charged ion at *m/z* 901.34 were extracted from the raw LC-MS/MS data. Glycan structures were assigned on the basis of the known MS/MS fragmentation patterns in negative-ion mode (78, 79, 80) and general glycobiological knowledge, with help of Glycoworkbench (81) and Glycomod (82) software. The B-, C-, Y-, and Z-type fragments were detected and described in the Figure 5.

### Extraction and HPTLC analysis of CHO-Lec2 neutral GSLs

Isolation, fractionation, and HPTLC analysis of CHO-Lec2 neutral GSLs were performed as described (1). Briefly, GSLs were extracted with chloroform/methanol, desalted and separated from phospholipids and gangliosides.

GSLs solubilized in chloroform/methanol (2:1, v/v) were applied to HPTLC plates (Kieselgel 60, Merck) and developed in chloroform/methanol/water (55:45:9, v/v/v). Dried plates were stained with orcinol or prepared for antibody assay. For that purpose, plates were immersed in 0.05% polyisobutylmethacrylate in hexane for 1 min, dried, soaked in TBS, and blocked in 5% bovine serum albumin in TBS for 1 h. After washing in TBS, plates were incubated overnight with human anti-P1 antibody diluted 1:50 in TBS/1% BSA, followed by incubation with anti-human biotinylated antibody (1:1000), ExtrAvidin-alkaline phosphatase conjugate (1:5000), and BCIP/NBT substrate solution.

### MALDI-TOF mass spectrometry analysis of neutral GSLs extracted from CHO-Lec2

MALDI-TOF mass spectrometry was carried out on an ultrafleXtreme MALDI TOF/TOF instrument (BrukerDaltonics), with the use of Peptide Calibration Standard (BrukerDaltonics) as external calibration and Norharmane (9H-Pyrido[3,4-b]indole; Sigma) as a matrix (10 mg/ml, chloroform:methanol, 2:1, v/v). GSLs were dissolved in chloroform/methanol (2:1, v/v). Spectra were scanned in the range of *m/z* 700–2000 in the reflectron-positive mode.

### Quantitative analysis of A4GALT transcript in CHO-Lec2 cells

mRNA was isolated from CHO-Lec2 cells using GeneMATRIX Universal RNA Purification Kit (EURx). The complementary DNAs (cDNAs) were synthesized using SuperScript III First-Strand Synthesis kit (Life Technologies) with oligo(dT) primers. Quantitative polymerase chain reaction (qPCR) was performed on 75 ng of cDNA on the 7500 Fast Real-Time PCR System (Life Technologies), according to the manufacturer’s instructions. The same custom TaqMan assay targeting human *A4GALT* was used as in (83). For endogenous control normalization, the custom assay targeting Chinese hamster *GAPDH* was used, with the following primers: forward (5’→3’) TGGAAAGCTTGTCATCAAC and reverse (5’→3’) GAAGACGCCAGTAGATTCC and probe (5’→3’) AGGCCATCACCATCTTCCAG.

### Flow cytometry

CHO-Lec2 cells were scraped, washed (all washes were done with PBS), and incubated for 30 min on ice with anti-P1 (clone P3NIL100; 1:400; all dilutions were done with 1% BSA in PBS), anti-P1 (clone 650; 1:200) antibodies, washed, and incubated with secondary FITC-conjugated antibodies: anti-human IgM (1:100), anti-mouse IgM (1:100). To analyze Shiga toxin binding, cells were incubated with 1 μg/ml Stx1B or Stx2B, then washed, and incubated with anti-6x-His Tag antibody (1:1000), followed by washing and incubation with FITC-conjugated anti-mouse IgG (1:100). To assess the level of Gb3 alone, cells were incubated with FITC-conjugated anti-CD77 (1:100). After incubation with FITC-conjugates cells were washed, resuspended in 500 μl of cold PBS, and subjected to flow cytometry analysis on FACSCalibur (BD Biosciences). In total, 1 × 10^5^ of events from the gated population were analyzed by Flowing Software (Perttu Terho, University of Turku).

Human RBCs received from 44 healthy donors (including 7 NOR-positive) were analyzed by flow cytometry. The same blood samples were previously used in the evaluation of anti-P1 (P3NIL100 and 650) and anti-NOR (nor118) binding (32). The study was approved by the Wrocław Medical University Bioethics Committee, Consent 641/2014, December 14, 2014. Blood from healthy individuals was obtained following an informed consent according to the Declaration of Helsinki. Whole blood was collected on EDTA and RBCs were subjected to the flow cytometry analysis during next 24 h, otherwise washed RBCs were stored in CellStab low-ionic-strength preservative solution (DiaMed, Cressier, Switzerland), and analyzed freshly after thawing. RBCs were washed five times with cold PBS, then 100 μl of 0.5% (v/v) cell suspension in PBS was incubated with an equal volume of 1 μg/ml Stx1B, followed by incubations with anti-6x-His Tag antibody (1:1000) and FITC-conjugated anti-mouse IgG (1:100). In total, 2.5 × 10^5^ of events from gated population recorded on FACSCalibur were analyzed by Flowing Software.

Antibody and Stx B subunits binding capacity per cell (CHO-Lec2 or human RBC) was determined with the use of Quantum (Bio-Rad) consisting of five populations of calibration microspheres. During each flow cytometry experiment, the fluorescence intensity of the Quantum beads was measured in order to plot the calibration curve (median fluorescence intensity versus Molecules of Equivalent Soluble Fluorochrome units). Antibody (or Stx B subunits) binding capacities were calculated by interpolation from the curve accordingly to the manufacturer’s protocol and the fluorophore-to-protein molar ratios of FITC-conjugates. Results of Stx1B binding to RBCs were presented on univariate scatter plot (84).

### Cytotoxicity assay

In total, 2 × 10^4^ CHO-Lec2 cells were seeded in 96-well plates (Wuxi NEST Biotechnology Co, Ltd) in complete DMEM/F12. After 24 h medium was replaced by 100 μl/well of serum-free DMEM/F12 containing 0.01, 0.05, 0.1, 0.5, 1 ng/ml of Stx1 or Stx2 holotoxins (all concentrations were run in triplicates). After 20 h of toxin treatment, 20 μl/well of MTS tetrazolium compound (CellTiter 96 AQ_ueous_ One Solution Assay, Promega) was added. Plates were incubated in humidified, 5% CO_2_ atmosphere for 2.5 h, then absorbance at 490 nm was recorded on ELISA plate reader. Background absorbance registered at zero cells/well was subtracted from the data and the absorbance of wells incubated in medium without addition of Stx was taken as 100% of cell viability. Each experiment was performed at least three times.

### Statistical analysis

For statistical analysis of Stx1B binding capacity per RBC quantified by flow cytometry using Quantum microspheres for different *A4GALT* genotypes, we performed a Mann–Whitney U-test. Cytotoxicity assays were analyzed using the *t*-test for independent groups with Holm–Bonferroni correction.

## Data availability

Raw MS data of N-glycans analyzed in the paper are available at the GlycoPOST, ID GPST000155 (https://glycopost.glycosmos.org/preview/4702278535fd87626d6369), as well as raw MS data of GSLs, ID GPST000156 (https://glycopost.glycosmos.org/preview/12959320725fd8767158705).

## Conflict of interest

The authors declare that they have no conflicts of interest with the contents of this article.
