# Supplementary material for: Human Gb3/CD77 synthase produces P1 glycotope-capped N-glycans, which mediate Shiga toxin 1 but not Shiga toxin 2 cell entry
Source: J Biol Chem. 2021 Jan 15;296:100299. doi: 10.1016/j.jbc.2021.100299 (PMC7949097; doi:10.1016/j.jbc.2021.100299)
Supplement: Table S1 and Figures S1 to S4 [file mmc1.pdf]

Supporting Information

Human Gb3/CD77 synthase produces P1 glycotope-capped N-glycans, which mediate Shiga toxin 1 but not Shiga toxin 2 cell entry

Katarzyna Szymczak-Kulus, Sascha Weidler, Anna Bereznicka, Krzysztof Mikolajczyk, Radoslaw Kaczmarek, Bartosz Bednarz, Tao Zhang, Anna Urbaniak, Mariusz Olczak, Enoch Y. Park, Edyta Majorczyk, Katarzyna Kapczynska, Jolanta Lukasiewicz, Manfred Wuhrer, Carlo Unverzagt, Marcin Czerwinski

Contents

Table S1. Specificity of anti-P1 and anti-CD77 antibodies employed in western blotting and flow cytometry analysis.

Figure S1. Western blotting analysis of CHO-Lec2 cell lysates.

Figure S2. Analysis of the top 15 most abundant N-glycans released from 0.5 x 10^6 CHO-Lec2 A4GALT Q211E cells on PGC nano-LC-ESI-MS/MS.

Figure S3. Comparison of Gb3 content and Gb3/CD77 synthase / p.Q211E mutein protein amount in CHO-Lec2 A4GALT and CHO-Lec2 A4GALT Q211E.

Figure S4. Flow cytometry analysis of anti-P1 (650) (A), anti-P1 (P3NIL100) (B) and Stx1B (C) binding to *P^1P^2* human RBCs, untreated and treated with papain.

Table S1. Specificity of anti-P1 and anti-CD77 antibodies employed in western blotting and flow cytometry analysis. Cer, ceramide; R, core N-glycan structure; GSL, glycosphingolipid; GP, glycoprotein.

Glycan structure	anti-P1		anti-CD77 (5B5)
	P3NIL100	650	
**Gb3** (Galα1→4Galβ1→4Glc-Cer)	-	+	+
**P1 GSL** (Galα1→4Galβ1→4GlcNAcβ1→3Galβ1→4Glc-Cer)	+	+	-
**P1 GP** (Galα1→4Galβ1→4GlcNAcβ1→R)	+	+	-

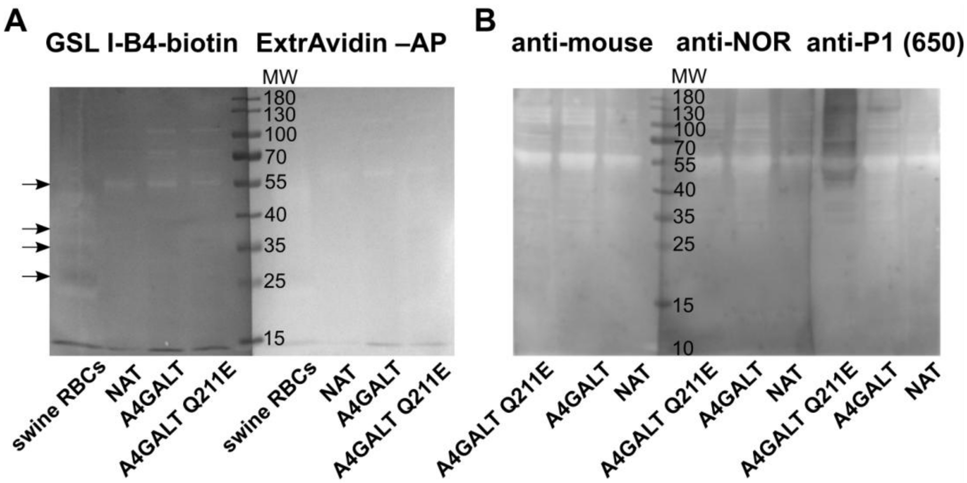

Figure S1. Western blotting analysis of CHO-Lec2 cell lysates. A) Analysis of CHO-Lec2 cell lysates stained with biotinylated GSL I-B4 lectin recognizing Galα1→3Gal structures. **B)** Analysis of CHO-Lec2 cell lysates stained with anti-P1 (650) and anti-NOR antibodies. Swine RBCs, lysates of swine RBC membranes used as the positive control; NAT, untransfected CHO-Lec2; A4GALT, CHO-Lec2 A4GALT; A4GALT Q211E, CHO-Lec2 A4GALT Q211E.

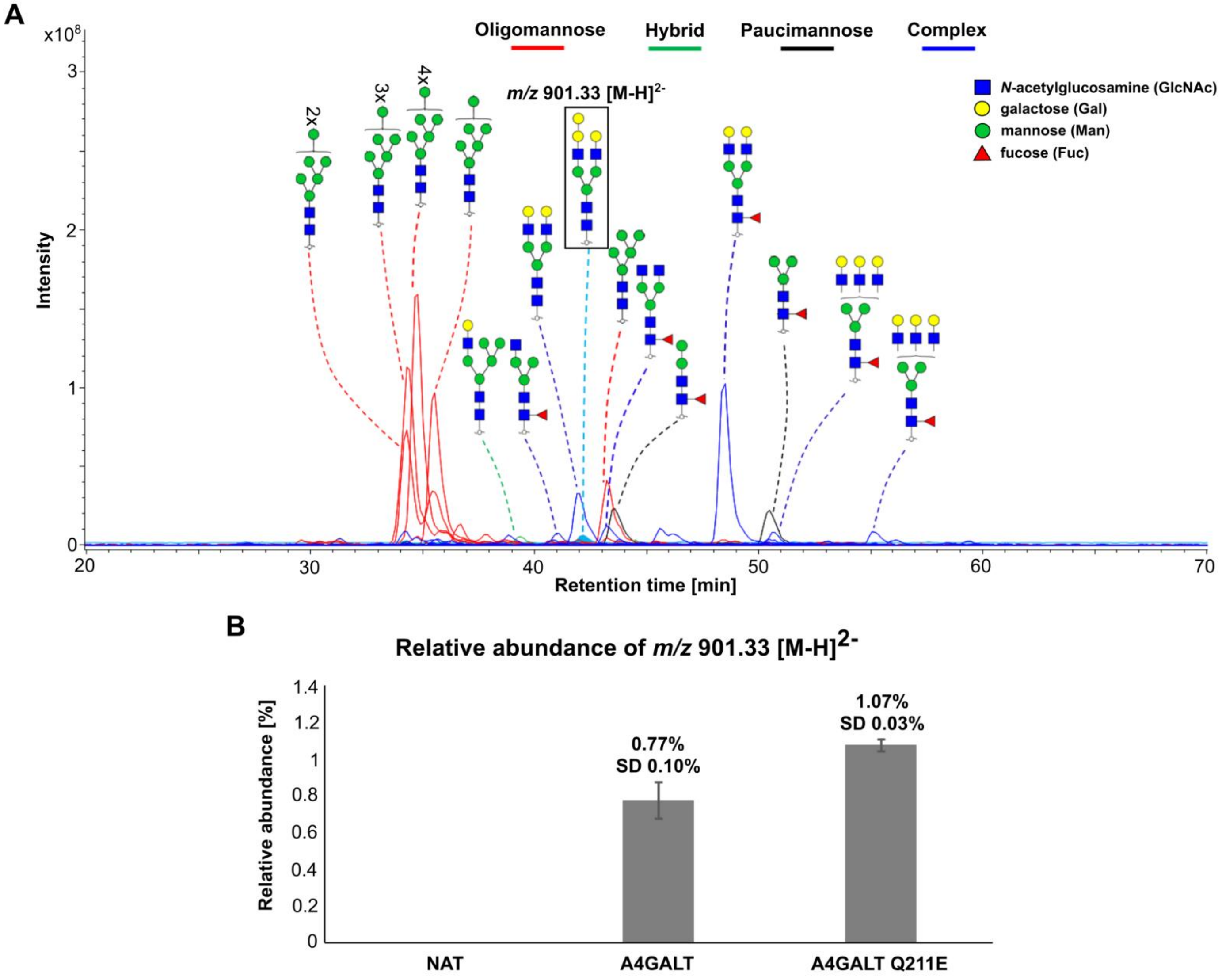

Figure S2. **Analysis of the top 15 most abundant N-glycans released from 0.5 x 10^6 CHO-Lec2 A4GALT Q211E cells on PGC nano-LC-ESI-MS/MS. A)** Combined extracted ion chromatograms (EICs) of top 15 N-glycans. **B)** Relative abundance of the N-glycan at *m/z* 901.33 $[M-H]^{2-}$ displayed as mean relative abundance with standard deviation (SD); N=3.

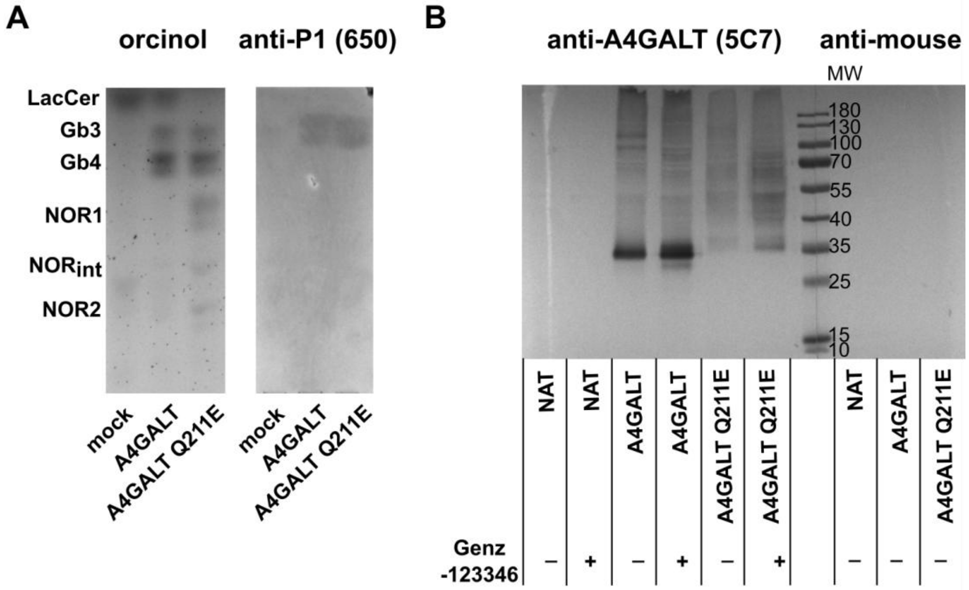

Figure S3. Comparison of Gb3 content and Gb3/CD77 synthase / p.Q211E mutein protein amount in CHO-Lec2 A4GALT and CHO-Lec2 A4GALT Q211E. A) HPTLC analysis of neutral glycosphingolipids purified from CHO-Lec2 cells stained with orcinol and anti-P1 (650). **B)** Western blotting analysis of CHO-Lec2 cell (cultured in presence or absence of Genz-123346) lysates stained with anti-A4GALT (5C7) antibody. Mock, CHO-Lec2 cells transduced with an empty lentivirus used as the negative control; NAT, untransfected CHO-Lec2; A4GALT, CHO-Lec2 A4GALT; A4GALT Q211E, CHO-Lec2 A4GALT Q211E.

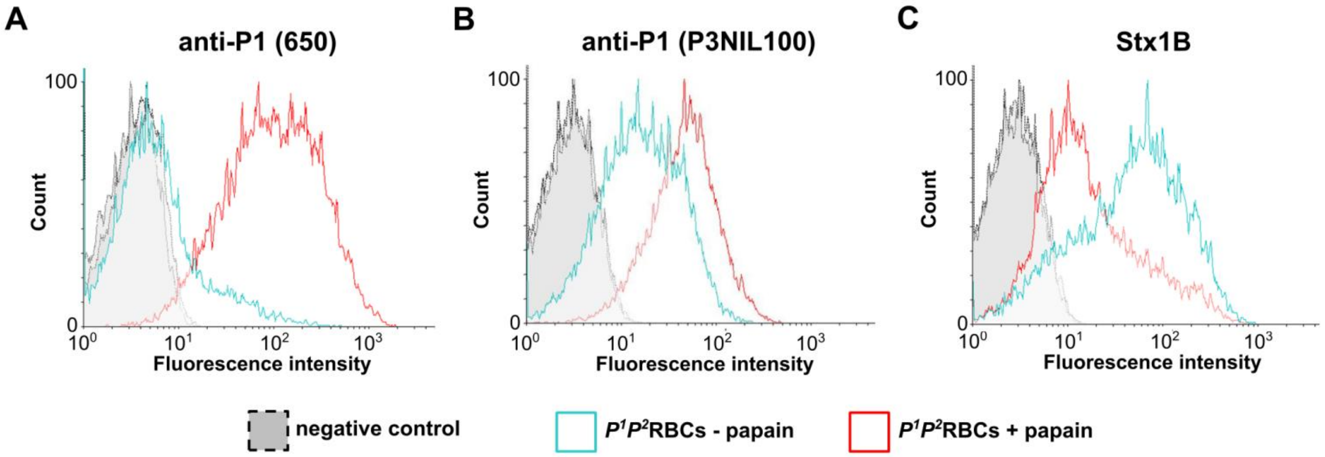

Figure S4. Flow cytometry analysis of anti-P1 (650) (A), anti-P1 (P3NIL100) (B) and Stx1B (C) binding to *P^1P^2* human RBCs, untreated and treated with papain.
